# Quantitative Evaluation of a New Posturo-Locomotor Phenotype in a Rodent Model of Acute Unilateral Vestibulopathy

**DOI:** 10.3389/fneur.2020.00505

**Published:** 2020-06-05

**Authors:** Guillaume Rastoldo, Emna Marouane, Nada El Mahmoudi, David Péricat, Audrey Bourdet, Elise Timon-David, Olivier Dumas, Christian Chabbert, Brahim Tighilet

**Affiliations:** ^1^Aix Marseille Université-CNRS, Laboratoire de Neurosciences Sensorielles et Cognitives, LNSC UMR 7260, Equipe Physiopathologie et Thérapie des Désordres Vestibulaires, Groupe de Recherche Vertige (GDR#2074), Marseille, France; ^2^Société Française de Kinésithérapie Vestibulaire, Lyon, France

**Keywords:** posture, locomotor activity, vestibular compensation, unilateral vestibular lesion, vestibular syndrome, behavior, ethovision

## Abstract

Vestibular pathologies are difficult to diagnose. Existing devices make it possible to quantify and follow the evolution of posturo-locomotor symptoms following vestibular loss in static conditions. However, today, there are no diagnostic tools allowing the quantitative and spontaneous analysis of these symptoms in dynamic situations. With this in mind, we used an open-field video tracking test aiming at identifying specific posturo-locomotor markers in a rodent model of vestibular pathology. Using Ethovision XT 14 software (Noldus), we identified and quantified several behavioral parameters typical of unilateral vestibular lesions in a rat model of vestibular pathology. The unilateral vestibular neurectomy (UVN) rat model reproduces the symptoms of acute unilateral peripheral vestibulopathy in humans. Our data show deficits in locomotion velocity, distance traveled and animal mobility in the first day after the injury. We also highlighted alterations in several parameters, such as head and body acceleration, locomotor pattern, and position of the body, as well as “circling” behavior after vestibular loss. Here, we provide an enriched posturo-locomotor phenotype specific to full and irreversible unilateral vestibular loss. This test helps to strengthen the quantitative evaluation of vestibular disorders in unilateral vestibular lesion rat model. It may also be useful for testing pharmacological compounds promoting the restoration of balance. Transfer of these novel evaluation parameters to human pathology may improve the diagnosis of acute unilateral vestibulopathies and could better follow the evolution of the symptoms upon pharmacological and physical rehabilitation.

## Introduction

Unilateral vestibular neurectomy (UVN) induces characteristic vestibular syndrome, composed of oculomotor, posturo-locomotor, and cognitive deficits in a rodent model. These vestibular disorders occur as a result of alterations in the vestibulo-oculomotor and vestibulo-spinal reflexes, as well as in vestibulo-cortical and vestibulo-cerebellar pathways. In humans, as in animals, vestibular syndrome can be split into several phases of different amplitudes depending on the type, stage, and severity of peripheral damage (1). In the UVN model, vestibular syndrome is particularly severe as a result of the massive imbalance in neuronal activity between the ipsilateral and contralateral vestibular nuclei (2, 3). The acute phase of vestibular syndrome in the UVN rodent model lasts several hours but may extend to days and is characterized by static and dynamic disorders (posturo-locomotor and oculomotor symptoms) in their most severe forms. This is followed by a partially compensated phase in which spontaneous resting activity recovers in neurons of the vestibular nuclei ipsilateral to the lesion. This leads to a full disappearance of the static disorders. However, some dynamic deficits remain poorly compensated and never fully disappear. These data are well-documented in several reviews (1, 4–10).

**Figure 1:**
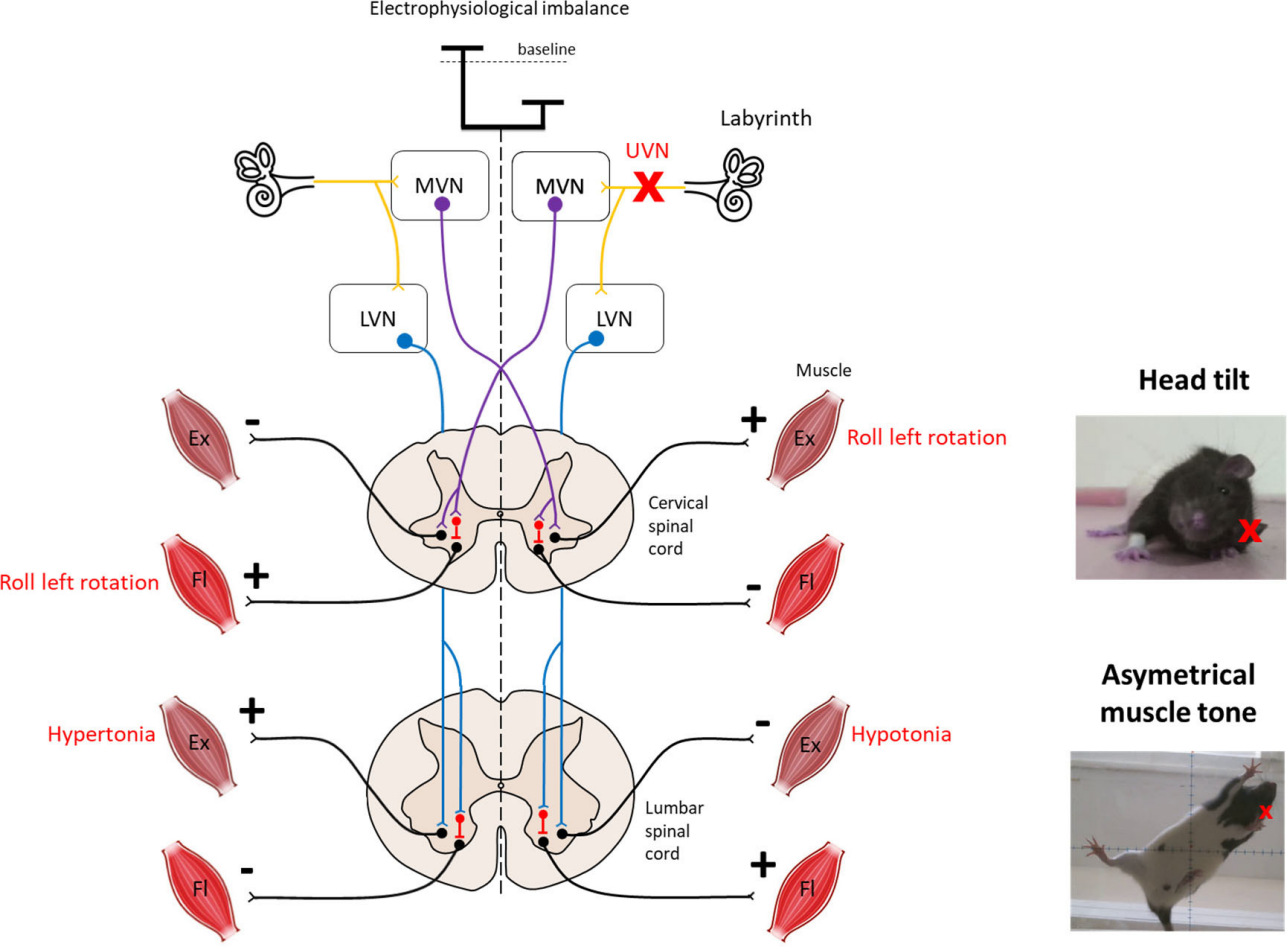
Overview of vestibulospinal reflexes to support the head tilt and hypotonia observed after unilateral vestibular neurectomy. The medial vestibulo-spinal tract (purple) is primarily composed of axons from the medial vestibular nucleus (MVN) and mainly projects contralaterally to the cervical spinal cord to mediate the vestibulocollic reflex. The lateral vestibulospinal tracts (blue) is primarily composed of axons from the lateral vestibular nucleus (LVN) and projects ipsilaterally to the entire length of the spinal cord to influence the extensor musculature of the body involved in balance. Both tracts exert excitatory effects on extensor motoneurons with some inhibitory effects on flexor motoneurons in normal condition. Left unilateral vestibular neurectomy (UVN) induces a electrophysiological imbalance between homologous vestibular nuclei. Loss of activity in the ipsilateral vestibular nucleus induces hypotonia of the lumbar extensor muscles via the lateral vestibulospinal tract. In contrast, the increase in activity in the contralateral lateral vestibular nucleus induces hypertonia of the extensor muscles opposite to the lesion as observed on the picture. Left UVN induces a head tilted (rolled) to the left side (see picture). Extensor activity is induced on the side to which the head is inclined, and flexor activity is induced on the opposite side via the medial vestibulospinal tract (11). Ex, Extensor muscle; Fl, Flexor muscle; LVN, Lateral vestibular nucleus; MVN, Medial vestibular nucleus.

The vestibular nuclei directly impact postural control through two major descending pathways to the spinal cord: the lateral and the medial vestibulospinal tracts (Figure 1). The medial vestibulospinal tract (MVST) is mainly composed of axons from the medial vestibular nucleus and, to a lesser extent, fibers from the lateral and descending (inferior) vestibular nucleus. This tract descends bilaterally and innervates the upper cervical regions of the spinal cord that innervate the upper-body musculature, particularly the neck musculature, essential for stabilizing the head in static or dynamic conditions (12–14). The lateral vestibulospinal tract (LVST) is composed mainly of axons from neurons in the lateral vestibular nucleus (Deiter's nucleus), with some contribution from the inferior vestibular nuclei. This tract descends ipsilaterally and innervates the entire length of the spinal cord, modulating the extensor musculature of the body. The LVST mainly terminates on interneurons in Rexed's laminae VII and VIII in the forelimb and hindlimb segments of the spinal cord. Rexed's VII laminae are also an important site for reticulospinal and corticospinal pathway termination. Thus, the vestibulospinal pathways are well-positioned to modulate the reflex responses to anticipated or imposed body displacements (15–17).

Current examinations of patients with vestibular loss generally involve assessment of the vestibulo-ocular reflex (VOR) (18) and vestibulo-spinal reflex with standing balance studies [(19–21), for review see Cohen (22)]. However, standing and walking are different motor functions. A patient's gait can be measured with a gyroscope system that records trunk sway (23) or with tests of walking balance such as the Tandem walking test (24, 25) or the 10-Meter Walk test (26). In most cases, walking balance tests are not useful for screening people for vestibular impairments but can be useful in vestibular rehabilitation in patients with known diagnoses (24). Among the large number of studies that have investigated postural and ocular reflex deficits after acute unilateral vestibular loss in rats, only a few have thoroughly investigated locomotor activity (27–29) (Porter et al., 1990). Furthermore, the analysis is restricted to velocity, distance traveled, or spatial exploration behavior. The present study was designed to further decipher new parameters for quantitative evaluation of posturo-locomotor syndrome and its compensation over time. We evaluated the spontaneous posturo-locomotor activity of the rat in an open field using up-to-date animal video tracking software and correlated these data with those obtained in a human clinic in vestibular pathology.

## Materials and Methods

### Animals and Experimental Protocols

Sixteen adult long evans rats (250–300 g) were used for this study. All experiments were performed in accordance with the National Institutes of Health's Guide for Care and Use of Laboratory Animals (NIH Publication no. 80-23) revised in 1996 for the UK Animals (Scientific Procedures) Act of 1986 and associated guidelines or the Policy on Ethics approved by the Society for Neuroscience in November 1989 and amended in November 1993 and under the veterinary and National Ethical Committee supervision (French Agriculture Ministry Authorization: B13-055-25). Present study was specifically approved by Neurosciences Ethic Committee N°71 from the French National Committee of animal experimentation. Every attempt was made to minimize both the number and the suffering of animals used in this experiment. Rats had free access to food and water and were housed individually under a constant 12 h light. Animals were divided into 2 groups as follow: a sham operated group (*n* = 8 female), a unilateral vestibular neurectomy (uvn) group (*n* = 4 male and *n* = 4 female).

### Unilateral Vestibular Neurectomy

Animals were submitted to a left-side vestibular nerve section (*n* = 8) following the surgical procedure previously reported in the literature (30). Thirty minutes after a subcutaneous injection of buprenorphine (Buprecare® 0.02 mg/kg), the rats were placed in the induction box and left for 5 min (isoflurane concentration 4%). Once they were deeply anesthetized, they were intubated and, during the surgery, the anesthesia was maintained at an isoflurane concentration of 3%. A tympanic bulla approach gave access to the vestibular nerve: the cervical muscular planes were dissected leading to the tympanic bulla, which was widely drilled to expose the stapedial artery and the promontory containing the cochlea. The cochlea was drilled, exposing the cochlear nerve. The cochlear nerve meatus was enlarged with a needle leading to the vestibulocochlear nerve, which was sectioned at its entry into the brainstem after aspiration of the Scarpa's ganglion. The wound was closed using a stapler. Before awakening the animal, a solution of Ringer Lactate (Virbac; 10 ml/kg) was administered subcutaneously in order to alleviate the dehydration resulting from the inability of the animal to drink normally as a result of the injury. For the sham operated group (*n* = 8), surgery was stopped at the opening of the tympanic bulla.

The successfulness of the surgery is attested at the behavioral level by the presence of a characteristic vestibular syndrome and at the histological level by the observation under optical microscopy of the full section of the 8th cranial nerve between Scarpa ganglion and vestibular nuclei from the brainstem [see Péricat et al. (30) for details].

### Open Field for Video Tracking

**Figure 2:**
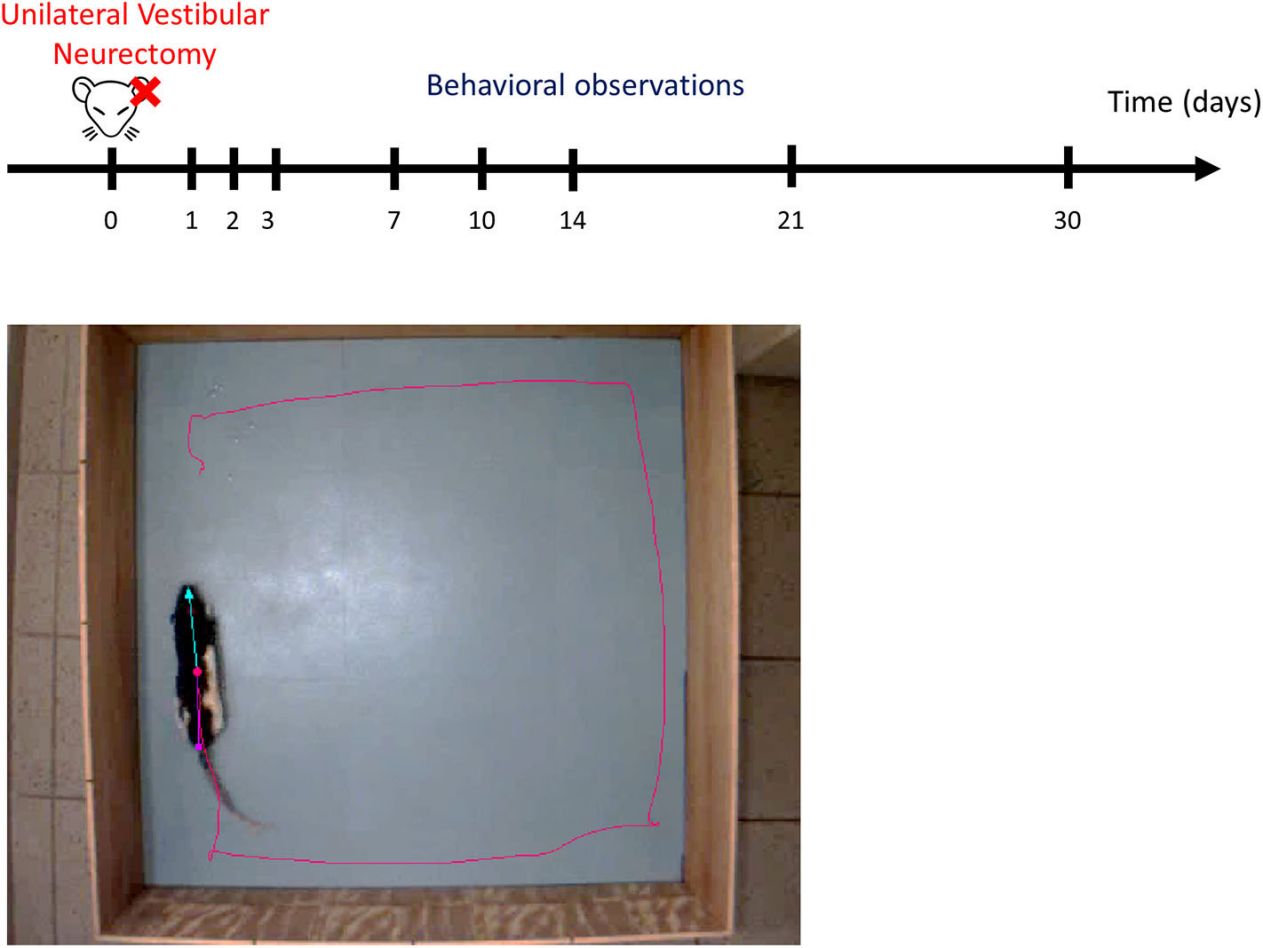
Experimental protocol. Upper part: schematic representation of behavioral observation in video-tracking to evaluate the time course of functional recovery at different post-UVN days. Behavioral analyses were made the day before the lesion, serving as a reference value, and then were performed at days (D) 1, 2, 3, 7, 10, 14, 21, and 30 post-lesion. Lower part: screenshot of the video recording used in the analysis. Ethovision™ automatically detect the following body points throughout the recordings: nose point (blue triangle), center point (red circle), and tail base (purple square) of each animal. The red line illustrates the plot of the center point while the animal is moving. Each animal allowed to explore the open field for 10 min. The open field is a square of 80 × 80 cm.

Animals were individually placed in an open field (80 × 80 × 40 cm). Their behavior was recorded for 10 min using a digital camera and analyzed with EthoVision™ XT 14 software (Noldus) (Figure 2). The surfaces of the open field were cleaned thoroughly between trials. To minimize stress, the room was lit as dimly as possible while allowing us to clearly discern the rats. At the beginning of the session, the rat was placed on the right side of the field, head in front of the wall. A first acquisition was done the day before the lesion, serving as a reference value, and then acquisitions were performed at days (D) 1, 2, 3, 7, 10, 14, 21, and 30 post-lesion.

### Detection and Behavioral Analysis

We used the dynamic subtraction method. Ethovision™ automatically detected the following body-points throughout the recordings: nose point, center point, and tail base of each animal (as shown in Figure 2 and in **Figures 6F** and **7F** for the recall).

We used 3 analysis profile for 19 variables we selected for analysis (Table 1). The first profile does not use a filter and all data were analyzed (No filter—All data). The second profile uses the minimal distance moved (MDM) smoothing method to filter out small movements (<0.7 cm) of the subject's center point that are caused by random noise and all data were analyzed (MDM—All data). The third profile uses the MDM smoothing method and analyzed only selected track segments while the animal is in motion (MDM—Moving).

**Table 1:** Variables measured with Ethovision XT 14.

**Variable**	**Body point**	**Filter—segment**	**Measurement**	**Unit**
Movement	Center point	No filter—all data	Duration not moving	%
Mobility state	Whole body	No filter—all data	Duration highly mobile	%
Mobility state	Whole body	No filter—all data	Duration mobile	%
Mobility state	Whole body	No filter—all data	Global mobility (sum of Highly mobile and Mobile)	%
Mobility state	Whole body	No filter—all data	Duration Immobile	%
Acceleration state (body)	Center point	No filter—all data	Frequency for High and Low Accelerations	
Acceleration state (head)	Nose point	No filter—all data	Frequency for High and low Accelerations	
Positive acceleration	Center point	No filter—all data	Mean	cm/s^2^
Negative acceleration	Center point	No filter—all data	Mean	cm/s^2^
Relative body angle	Center, nose and tail base point	MDM—all data	Mean	°
Velocity (head)	Nose point	MDM—all data	Mean and Maximum	cm/s
Cephalic nystagmus (bobbing)	Nose point	MDM—all data	Frequency of velocity for the nose point ≥ 100 cm/s	
Distance moved	Center point	MDM—when moving	Total	cm
Velocity (body)	Center point	MDM—when moving	Mean and Maximum	cm/s
Body axis rotation CW	Center to nose point	MDM—when moving	Frequency	
Body axis rotation CCW	Center to nose point	MDM—when moving	Frequency	
Body points rotation CW	Center point	MDM—when moving	Frequency	
Body points rotation CCW	Center point	MDM—when moving	Frequency	
Absolute meander	Center point	MDM—when moving	Mean	°/cm

*CW, clockwise (contralesional)°; CCW, counterclockwise (ipsilesional); MDM, Minimal Distance Moved*.

“Duration not moving” of rats was calculated with an average interval of 3 samples and a threshold of 2.00 cm/s for start and 1.75 cm/s for stop velocity. “Duration highly mobile, mobile and immobile” were calculated with an average interval of 3 samples and a threshold for highly mobile above 5%, mobile between 1 and 5% and immobile below 1%. “Global mobility” is the sum of the % of highly mobile and mobile. “Frequency for high and low acceleration” were calculated with an average interval of 3 samples with a threshold for high acceleration above 50 cm/s^2^ and exclude instances shorter than 0.20 s. “Mean positive and negative accelerations” were calculated by selecting with a filter only acceleration above 0 cm/s^2^ or under 0 cm/s^2^ on the data. Velocity for the nose point and center point was calculated with an average interval of 3 sample. “Multi condition” with the variable: “Velocity” for the nose point ≥100 cm/s (averaged over 3 samples) allow us to calculate the frequency of velocity for the nose point ≥100 cm/s. Velocity for the nose point ≥100 cm/s was associated with a cephalic nystagmus behavior when recorded. To select the track segments when the animal is in motion, we selected for every trial recorded in EthoVision™ the track-segments where the animal was moving (thresholds of 2.00 cm/s for start and 1.75 cm/s for stop velocity). “Body axis rotation” has been set to count every 1 rotation with a threshold of 30° allowing us to calculate when the animal is spinning around its own axis. “Body point rotation” has been set to count every 1 rotation with a threshold of 50° with a minimum distance moved of 2 cm allowing us to calculate the rotation in the open field when the animal walks around in circle.

### Statistical Analysis

The statistical analyses were evaluated by one-way repeated-measures ANOVA followed by a simple contrast to compare the postoperative time with the pre-operative time for each group (JASP). Difference between the Sham and NVU group were evaluated by two-way repeated measures ANOVA. If significant effect were found, *post-hoc* Bonferroni was performed (GraphPad, Prism). Results were considered significant at *p* < 0.05.

## Results

### Effect of UVN on Rat Activity and Spatial Exploration

**Figure 3:**
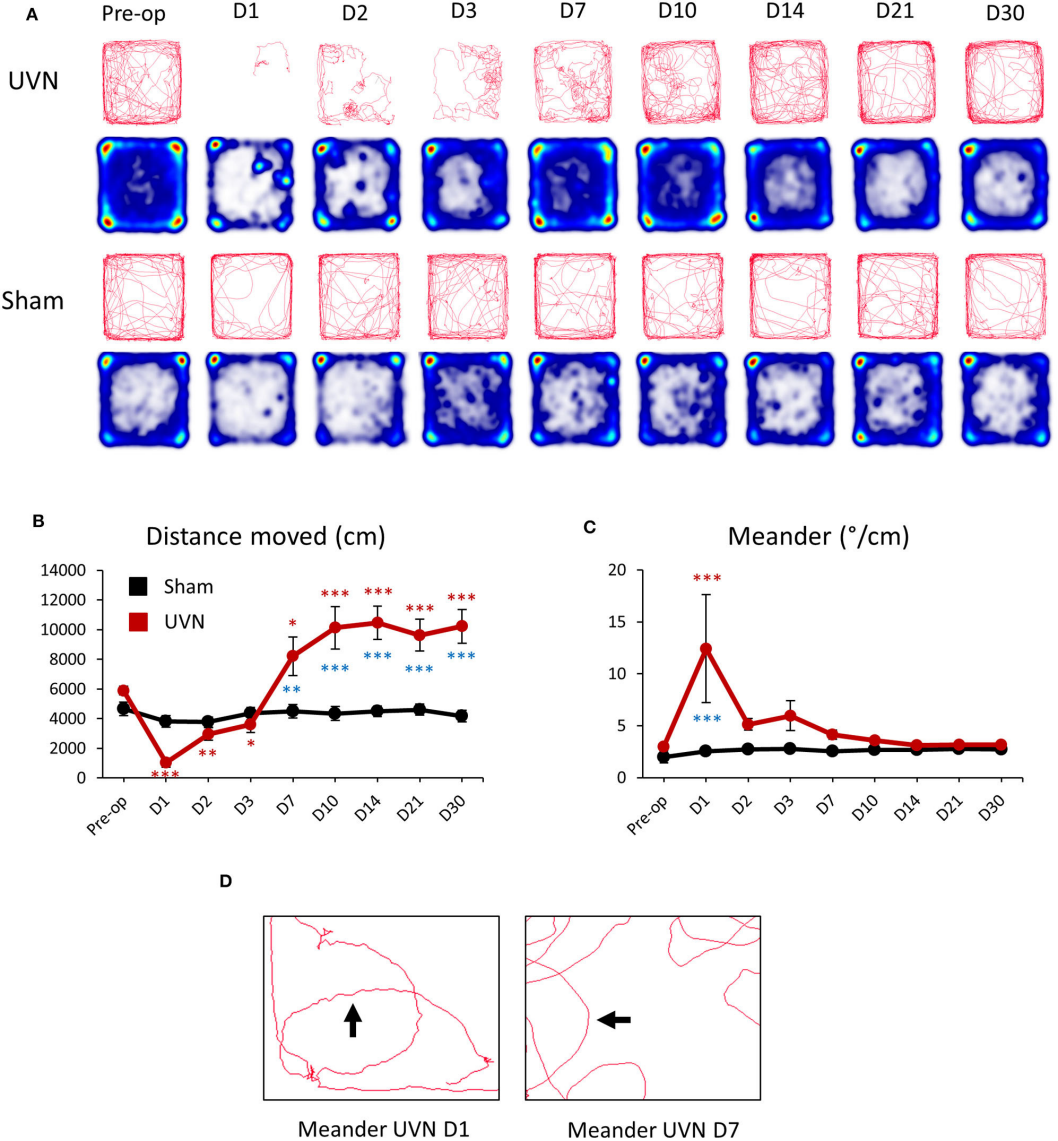
Effect of UVN on rat activity and spatial exploration in an open field. **(A)** Movement tracing and spatial exploration pattern (Heatmaps) of UVN and Sham rats in an open field over the 10 min analysis period. Heatmaps indicate the cumulative time at place (blue to red scale increasing duration). **(B)** Curve illustrating the mean postoperative recovery of the distance moved by rats on the open-field in the two experimental group of rats (Sham in black and UVN in red). **(C)** Curve illustrating the mean postoperative recovery of meander by rats on the open-field in the two experimental group of rats (Sham in black and UVN in red). Data represent mean ± SEM; *P < 0.05, **P < 0.01, ***P < 0.001. **(D)** Zoom on the movement tracing of a UVN rat 1 and 7 days after the surgery. Meander is defined as “the change in direction of movement of a subject relative to the distance moved by that subject and provides an indication of how convoluted the subject's trajectory is.” As observed on the movement tracing by rats 1 day after the surgery the trajectory (arrow) of the animal is more “tortuous” compared to the trajectory of the same rat seven days after UVN. A significant difference from the pre-operative value is indicated by * in black for the SHAM group. A significant difference from the pre-operative value is indicated by * in red for the UVN group. A significant difference between the SHAM and the UVN group is indicated with * in blue.

Unilateral vestibular neurectomy (UVN) produced significant changes in posturo-locomotor activity of rats in the open field (Figure 3A). UVN rats showed significant decreased in the total distance moved the first 3 days after UVN (Preop: 5897.88 ± 303.68; Day 1: 1,034 ± 308, *p* < 0.001; Day 2: 2,969 ± 419, *p* < 0.01; Day 3: 3,601 ± 530, *p* < 0.05). From day 7 until day 30, the mean distance traveled by lesioned rats was significantly increased relative to that observed before UVN (Day 7: 8,209 ± 1,314, *p* < 0.05; Day 10: 10,127 ± 1,418, *p* < 0.001; Day 14: 10,475 ± 1,125, *p* < 0.001; Day 21: 9,633 ± 1,081, *p* < 0.001; Day 30: 10,222 ± 1,141, *p* < 0.001; Figure 3B) and significantly differed from the Sham group (Day 7: *p* < 0.01; Day 10: *p* < 0.001; Day 14: *p* < 0.001; Day 21: *p* < 0.001; Day 30: *p* < 0.001). The average distance moved of rat from the Sham group was not significantly different to that observed before the sham lesion.

Meander, defined as tortuous/winding movement (Figure 3D) was significantly increased at day 1 (Preop: 2.98 ± 0.1; Day 1: 12.42 ± 5.19, *p* < 0.001) post-lesion and was significantly different from the Sham group (Day 1: *p* < 0.001). From day 2 until day 30, the meander of lesioned rats was no longer significantly different to that observed before UVN (Figure 3C). The meander of the Sham group was not significantly different to that observed before the sham lesion.

### Mobility of UVN and Sham Rats

**Figure 4:**
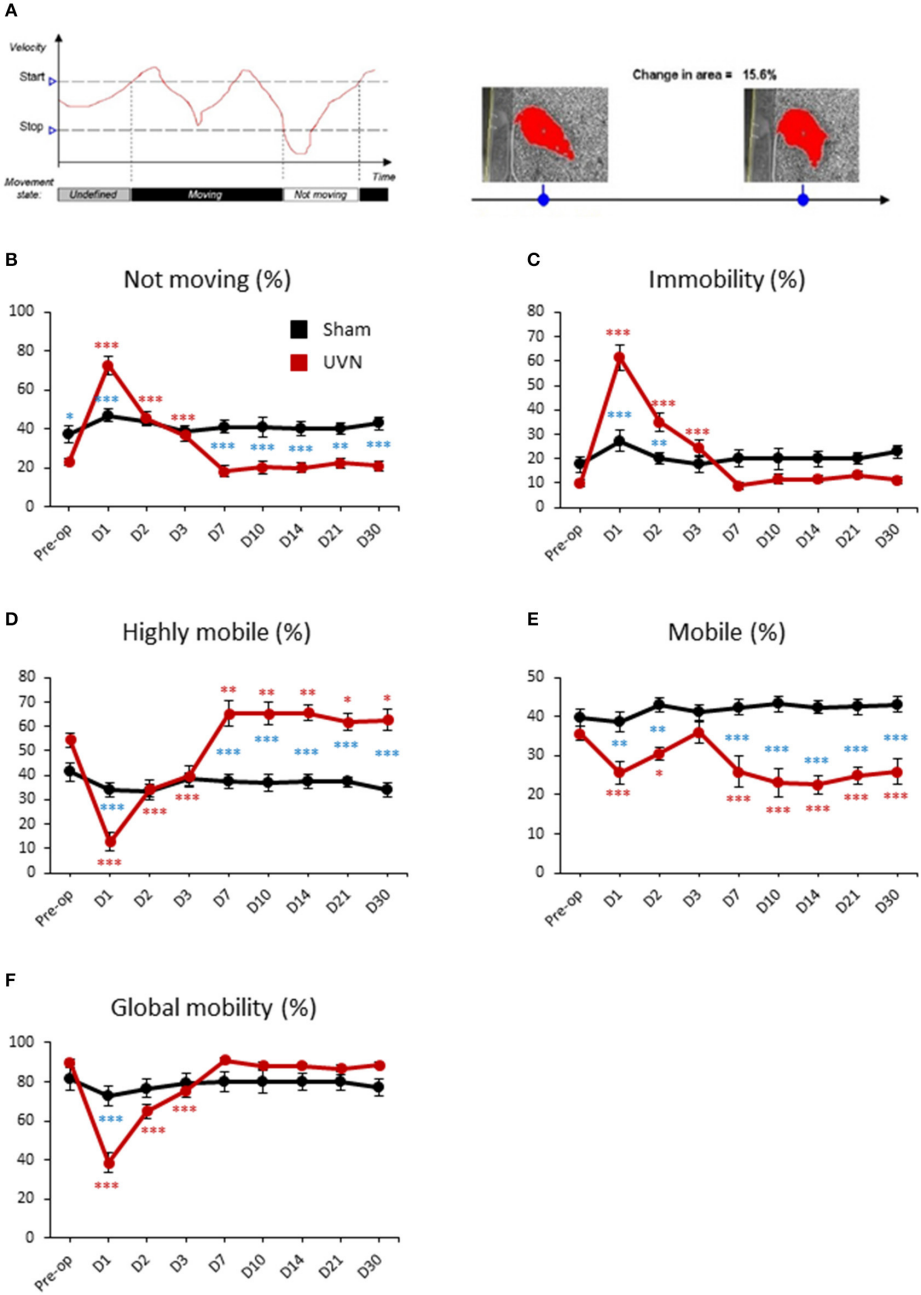
Mobility of UVN and Sham rats in the open field. **(A)** Illustration of the variable movement (on left) and mobility (on right). In the following example for movement, because the velocity initially lies between the Stop velocity and the Start velocity, the state is undefined. When velocity exceeds the Start velocity value, Movement is given the value “Moving.” When velocity drops below the Stop velocity value, Movement is given the value “Not moving.” Mobility can be defined as the degree of movement of an animal's body independent of spatial displacement of the center or any other body point, which is measured by Movement. The calculation of mobility does not use the x,y coordinates of the animal. Mobility is calculated 100% independent of movement of the coordinates identified as the center-point (or the nose/tail point). **(B)** Curves illustrating the kinetics of the % of time when the animal is not moving for UVN (red) and Sham (black) group. **(C)** Curves illustrating the kinetics of the % of time when the animal is immobile for UVN (red) and Sham (black) group. **(D)** Curves illustrating the kinetics of the % of time when the animal is highly mobile for UVN (red) and Sham (black) group. **(E)** Curves illustrating the kinetics of the % of time when the animal is mobile for UVN (red) and Sham (black) group. **(F)** Curves illustrating the kinetics of the % of time when the animal is either mobile or highly mobile (global mobility) for UVN (red) and Sham (black) group. Data represent mean ± SEM; *P < 0.05, **P < 0.01, ***P < 0.001. A significant difference from the pre-operative value is indicated by * in black for the SHAM group. A significant difference from the pre-operative value is indicated by * in red for the UVN group. A significant difference between the SHAM and the UVN group is indicated with * in blue.

The software discriminates between movement and mobility. Movement is a discrete variable, related to the center-point, with two possible states: “Moving” and “Not moving” (Figure 4A). The state “Moving” relates to states when running mean velocity exceeds 2.00 cm/s (start velocity). This state remains until the running mean velocity drops below 1.75 cm/s (stop velocity). It becomes “Not moving” until the running mean velocity reaches the start velocity again. Mobility can be defined as the degree of movement of an animal's body and is calculated 100% independent of movement of the coordinates identified as the center-point (or the nose/tail point).

The % of time not moving or in immobility of UVN rats followed the same time course. It increased significantly the first 3 days after UVN with a maximum value at day 1 (Preop: 23.09 ± 2.06; Day 1: 72.83 ± 4.69, *p* < 0.001; Day 2: 45.41 ± 3.78, *p* < 0.001; Day 3: 36.92 ± 3.36, *p* < 0.001; for % of time not moving and, Preop: 9.86 ± 1.39; Day 1: 61.56 ± 5.18, *p* < 0.001; Day 2: 35.09 ± 3.85, *p* < 0.001; Day 3: 24.37 ± 3.33, *p* < 0.001; for % of time in immobility) and returned to control values from day 7 until day 30 (Figures 4B,C). Interestingly, the difference between UVN and Sham groups differed for the % of time not moving or in immobility. Indeed, the % of time not moving was significantly different at pre-operative time (Pre-op: *p* < 0.05) and at days 1 (Day 1: *p* < 0.001), 7 (Day 7: *p* < 0.001), 10 (Day 10: *p* < 0.001), 14 (Day 14: *p* < 0.001), 21 (Day 21: *p* < 0.001), and 30 (Day 30: *p* < 0.001). Conversely, the % of time in immobility, which was not significantly different at pre-operative time, significantly differed at day 1 (Day 1: *p* < 0.001) and day 2 (Day 2: *p* < 0.01). These differences are due to the method of calculation of these two parameters (see above).

Mobility of the animal can be separated in % of time highly mobile (above 5% of change in area detected) and in % of time mobile (between 1 and 5% of change). The % of time highly mobile of UVN rats is the mirror curve of the % of time in immobility: decreased significantly the first 3 days after UVN with a maximum at day 1 (Preop: 54.44 ± 2.87; Day 1: 12.65 ± 3.71, *p* < 0.001; Day 2: 34.29 ± 4.06, *p* < 0.001; Day 3: 39.59 ± 4.25, *p* < 0.001) but was significantly increased from day 7 until day 30 (Day 7: 65.18 ± 5.16, *p* < 0.01; Day 10: 65.21 ± 4.71, *p* < 0.01; Day 14: 65.62 ± 3.44, *p* < 0.01; Day 21: 61.87 ± 3.61, *p* < 0.05; Day 30: 62.73 ± 4.41, *p* < 0.05). The % of time highly mobile between the Sham and the UVN group was significantly different on days 1, 7, 10, 14, 21, 30 (D1: *p* < 0.001; D7: *p* < 0.001; D10: *p* < 0.001; D14: *p* < 0.001; D21: *p* < 0.001; D30: *p* < 0.001; Figure 4D).

The % of time mobile of UVN rats decreased significantly at day 1 and day 2 (Preop: 35.68 ± 1.79; Day 1: 25.75 ± 2.91, *p* < 0.01; Day 2: 30.60 ± 1.67, *p* < 0.01) and then return to control value at day 3 but decreased once again from day 7 to day 30 (Day 7: 25.88 ± 3.97, *p* < 0.01; Day 10: 23.08 ± 3.46, *p* < 0.001; Day 14: 22.64 ± 2.31, *p* < 0.001; Day 21: 24.92 ± 2.05, *p* < 0.001; Day 30: 25.85 ± 3.22, *p* < 0.001). The same results was obtained when we compare the Sham and the UVN group (D1: *p* < 0.01; D2: *p* < 0.01; D7: *p* < 0.001; D10: *p* < 0.001; D14: *p* < 0.001; D21: *p* < 0.001; D30: *p* < 0.001; Figure 4E).

Global mobility (sum of highly mobile and mobile) of UVN rats significantly decreased the first 3 days after UVN (Preop: 91.12 ± 4.66; Day 1: 38.41 ± 5.18, *p* < 0.001; Day 2: 64.90 ± 3.85, *p* < 0.001; Day 3: 75.61 ± 3.31, *p* < 0.001) and then returned to control values from day 7 until day 30 (Figure 4F). Interestingly, the sham and the UVN groups significantly differed at day 1 (D1: *p* < 0.001) only.

For all parameters analyzed, the Sham group did not significantly differ from that observed before the sham lesion.

### Rotations Frequencies of UVN and Sham Rats

**Figure 5:**
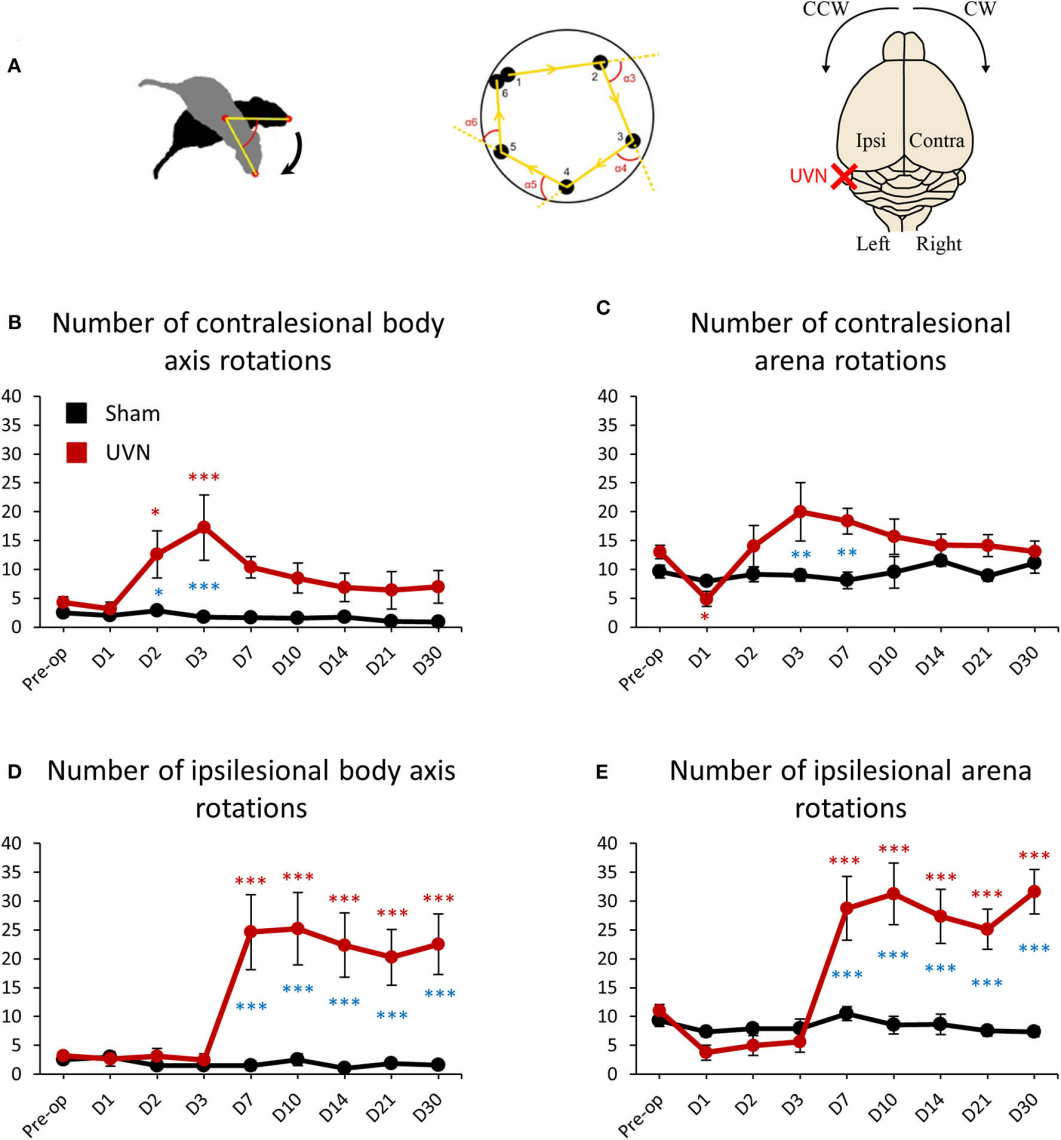
Rotation frequencies of UVN and Sham rats in the open field. **(A)** Illustration of body axis rotation (left part), arena rotation (middle part) and illustration of the contralateral and ipsilateral hemispheres regarding the lesion and turning direction (right part). A rotation in a counterclockwise (CCW) direction is a ipsilesional rotation and vice versa for clockwise (CW) rotations. Body axis rotation is a method used to quantify if the rat is spinning around its own axis. Arena rotation is suitable for when animal walks around in circles (middle part). **(B)** Curves illustrating the kinetics of body axis rotation frequencies on the intact side (contralesional rotations) of UVN (red) and Sham (black) group. **(C)** Curves illustrating the kinetics of arena rotation frequencies on the intact side (contralesional rotations) of UVN (red) and Sham (black) group. **(D)** Curves illustrating the kinetics of body axis rotation frequency on the lesioned side (ipsilesional rotation) of UVN (red) and Sham (black) group. **(E)** Curves illustrating the kinetics of Arena rotation frequency on the lesioned side (ipsilesional rotations) of UVN (red) and Sham (black) group. Data represent mean ± SEM; *P < 0.05, **P < 0.01, ***P < 0.001. A significant difference from the pre-operative value is indicated by * in black for the SHAM group. A significant difference from the pre-operative value is indicated by * in red for the UVN group. A significant difference between the SHAM and the UVN group is indicated with * in blue.

Animal's rotations were analyzed through monitoring both the rotations of rats around their body axis and the circling travel in the arena (Figure 5A). The animal's rotations can be analyzed in body axis rotation and in arena rotation. Body axis rotation quantifies if the rat is spinning around its own axis while arena rotation quantifies if the rat walks around the arena in circle (Figure 5A). Before the lesion UVN rats rotated 4.25 ± 0.97 times on themselves (body axis rotation) on the clockwise (contralesional) direction and 3.25 ± 0.55 times on the counterclockwise (ipsilesional) direction. After UVN the mean number of contralesional body axis rotation increased significantly at day 2 and 3 (Day 2: 12.62 ± 4.09, *p* < 0.05; Day 3: 17.25 ± 5.64, *p* < 0.001). From day 7 until day 30 the mean number of contralesional body axis rotations of UVN rats was no longer significantly different to that observed before the lesion. The Sham group rotated on themselves (body axis rotation) significantly less on the contralesional side compare to the UVN group at day 2 and day 3 (Day 2: *p* < 0.05; Day 3: *p* < 0.001). Similar results were obtained for the ipsilesional body axis rotation from day 7 to day 30 compare to the UVN group (Day 7: *p* < 0.001; Day 10: *p* < 0.001; Day 14: *p* < 0.001; Day 21: *p* < 0.001; Day 30: *p* < 0.001; Figure 5B). Conversely, the mean number of ipsilesional body axis rotation after UVN increased significantly from day 7 until day 30 (Day 7: 24.62 ± 6.49, *p* < 0.001; Day 10: 25.19 ± 6.27, *p* < 0.001; Day 14: 22.37 ± 5.54, *p* < 0.001; Day 21: 20.25 ± 4.79, *p* < 0.001; Day 30: 22.5 ± 5.24, *p* < 0.001; Figure 5D). The number of body axis rotation in either ipsilesional or contralesional side was not significantly affected by the sham surgery nor the postoperative time.

Before the lesion, UVN rats rotated 13 ± 1.13 times in the arena on the clockwise (contralesional) direction and 11 ± 1.06 on the counterclockwise (ipsilesional) direction. One day after UVN the mean number of arena rotations on the contralesional direction decreased significantly to 4.87 ± 1.28 (*p* < 0.05) and decreased insignificantly to 3.75 ± 1.30 (*p* = 0.115) on the ipsilesional direction due to the incapacity of UVN rats to move properly 1 day after the lesion. From day 2 until day 30, arena rotations of UVN rats on the contralesional direction did not significantly differed relative to that observed before the lesion. However, the contralesional arena rotations of the Sham group were significantly reduced at day 3 and 7 compared to the UVN group (Day 3: *p* < 0.01; Day 7: *p* < 0.01; Figure 5C). Conversely, the ipsilesional arena rotations of UVN rats was not affected by the lesion the first 3 postoperative days but increased significantly from day 7 to day 30 (Day 7: 28.75 ± 5.54, *p* < 0.001; Day 10: 31.25 ± 5.32, *p* < 0.001; Day 14: 27.37 ± 4.66, *p* < 0.001; Day 21: 25.12 ± 3.50, *p* < 0.001; Day 30: 31.62 ± 3.85, *p* < 0.001). Furthermore, the ipsilesional arena rotations of the UVN group were significantly increased from day 7 to day 30 compared to the Sham group (Day 7: *p* < 0.001; Day 10: *p* < 0.001; Day 14: *p* < 0.001; Day 21: *p* < 0.001; Day 30: *p* < 0.001; Figure 5E).

The number of arena rotations in either ipsilesional or contralesional side was not significantly affected by the sham surgery nor postoperative time for the Sham group.

### Locomotor Velocity of UVN and Sham Rats

**Figure 6:**
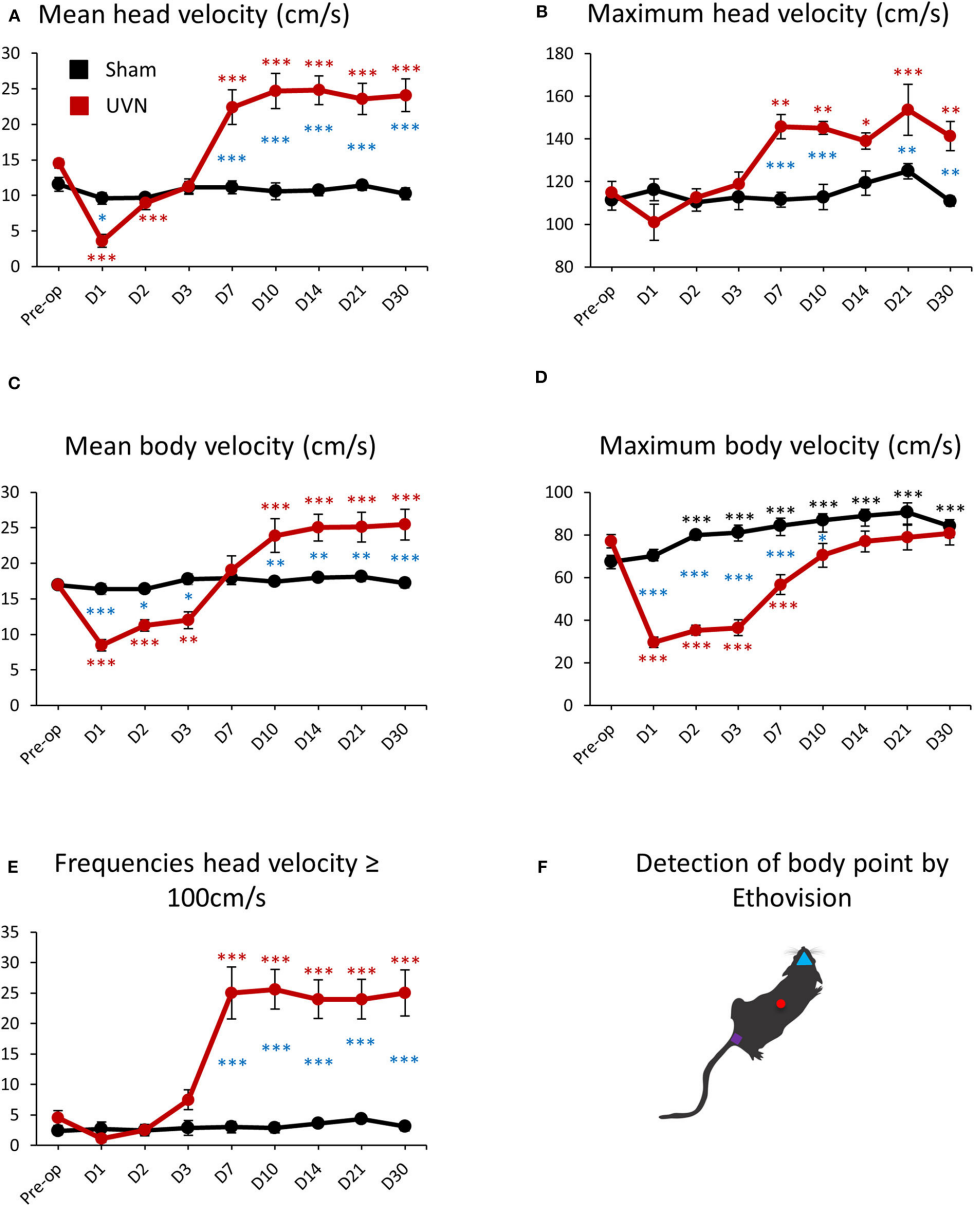
Locomotor velocity of UVN and Sham rats in the open field. **(A)** Curves illustrating the kinetics of the mean velocity (cm/s) of the head (calculated from the nose point) for UVN (red) and Sham (black) group. **(B)** Curves illustrating the kinetics of the maximum velocity (cm/s) of the head for UVN (red) and Sham (black) group. **(C)** Curves illustrating the kinetics of the mean velocity (cm/s) of the body (calculated from the center-point) for UVN (red) and Sham (black) group. **(D)** Curves illustrating the kinetics of the maximum velocity (cm/s) of the body for UVN (red) and Sham (black) group. **(E)** Curves illustrating the kinetics of the frequencies of the velocity of the head superior or equal to 100 cm/s, linked to the bobbing behavior (cephalic nystagmus) for UVN (red) and Sham (black) group. Bobbing behavior is defined when the velocity for the nose point is ≥ 100 cm/s. **(F)** illustration of the detection of body point by Ethovision™ throughout the recordings: nose point (blue triangle), center point (red circle), and tail base (purple square) of each animal. Data represent mean ± SEM; *P < 0.05, **P < 0.01, ***P < 0.001. A significant difference from the pre-operative value is indicated by * in black for the SHAM group. A significant difference from the pre-operative value is indicated by * in red for the UVN group. A significant difference between the SHAM and the UVN group is indicated with * in blue.

The mean velocity of the head (calculated from the nose-point) and the body (calculated from the center-point) of UVN rats was 14.50 ± 0.61 cm/s and 17 ± 0.53 cm/s before UVN. These values significantly decreased at day 1 and day 2 for the head velocity (Day 1: 3.61 ± 0.91, *p* < 0.001; Day 2: 8.94 ± 0.93, *p* < 0.001) and over the first 3 days after lesion for the body velocity (Day 1: 8.45 ± 0.80, *p* < 0.001; Day 2: 11.27 ± 0.79, *p* < 0.001; Day 3: 12 ± 1.17, *p* < 0.001). From day 7 until day 30, the mean head velocity of the UVN rats significantly increased (Day 7: 22.41 ± 2.43, *p* < 0.001; Day 10: 24.68 ± 2.46, *p* < 0.001; Day 14: 24.82 ± 2.02, *p* < 0.001; Day 21: 23.54 ± 2.19, *p* < 0.001; Day 30: 24.08 ± 2.29, *p* < 0.001). On the other hand, the mean velocity of the body of UVN rats was significantly increased from day 10 to day 30 (Day 10: 23.90 ± 2.38, *p* < 0.001; Day 14: 25.02 ± 1.90, *p* < 0.001; Day 21: 25.11 ± 2.08, *p* < 0.001; Day 30: 25.44 ± 2.13, *p* < 0.001; Figures 6A,C).

The mean velocity of the head or the body was not significantly affected by the sham surgery nor postoperative time for the Sham group.

Maximum head and body velocities during vestibular compensation evolved differently than the mean velocity. Maximum head velocity of UVN rats was not impaired after UVN during the first 3 days but increased significantly from day 7 to day 30 compare to pre-operative values (Preop: 114.83 ± 5.23; Day 7: 145.78 ± 5.67, *p* < 0.01; Day 10: 145.09 ± 3.03, *p* < 0.01; Day 14: 138.99 ± 3.85, *p* < 0.05; Day 21: 153.62 ± 11.90, *p* < 0.001; Day 30: 141.40 ± 6.78, *p* < 0.01; Figure 6B). Conversely, maximum body velocity of UVN rats was 77.02 ± 3.20 cm/s before UVN and significantly decreased with a peak at day 1 (29.64 ± 2.39, *p* < 0.001) until day 7 post-lesion (Day 2: 35.29 ± 2.26, *p* < 0.001; Day 3: 36.47 ± 3.79, *p* < 0.001; Day 7: 56.69 ± 4.70, *p* < 0.001). From day 10 until day 30, the maximum body velocity of the lesioned rats was no longer significantly different to that observed before UVN (Figure 6D). Maximum head velocity of the UVN group was significantly increased compared to the Sham group at day 7, 10, 21 and 30 post-lesion (Day 7: *p* < 0.001; Day 10: *p* < 0.001; Day 21: *p* < 0.01; Day 30: *p* < 0.01) and the maximum head velocity was not significantly affected by the sham surgery nor postoperative time (Figure 6B). Conversely, the maximum body velocity of the Sham group was significantly increased from day 2 to day 30 post-lesion compared to the pre-operative value (Day 2: 79.94 ± 2.89, *p* < 0.001; Day 3: 81.01 ± 3.52, *p* < 0.001; Day 7: 84.37 ± 3.48, *p* < 0.001; Day 10: 87 ± 2.83, *p* < 0.001; Day 14: 88.91 ± 3.01, *p* < 0.001; Day 21: 90.59 ± 4.39, *p* < 0.001; Day 30: 84.18 ± 3.05, *p* < 0.001, Figure 6D). The maximum body velocity of UVN rats was significantly decreased compared to the Sham group during the first 10 days post-lesion (Day 1: *p* < 0.001; Day 2: *p* < 0.001; Day 3: *p* < 0.001; Day 7: *p* < 0.001; Day 10: *p* < 0.05).

The increased head velocity from day 7 to day 30 for the UVN group was correlated with the appearance of cephalic nystagmus (bobbing) induced by the lesion (Video 1). Indeed, we could quantify the frequencies of bobbing with a high-pass filter to calculate the number of times the head (nose point) velocity exceeds 100 cm/s. As shown on Figure 6E, in physiologic condition, the software detected a few numbers of times when the head velocity exceeded 100 cm/s (Preop: 4.5 ± 1.21) because the rat can reach these values without being associated to bobbing behavior. However, the numbers of times for which the head velocity exceeded 100 cm/s did not change (between 2 and 4 detection) for the Sham group over time but bobbing frequencies on the UVN group increased significantly from day 7 to day 30 post-lesion (Day 7: 25 ± 4.25, *p* < 0.001; Day 10: 25.62 ± 3.24, *p* < 0.001; Day 14: 24 ± 3.16, *p* < 0.001; Day 21: 24 ± 3.24, *p* < 0.001; Day 30: 25 ± 3.79, *p* < 0.001; Figure 6E) and was correlated with the increased of maximum head velocity (see Figure 6B).

### Acceleration of UVN and Sham Rats

**Figure 7:**
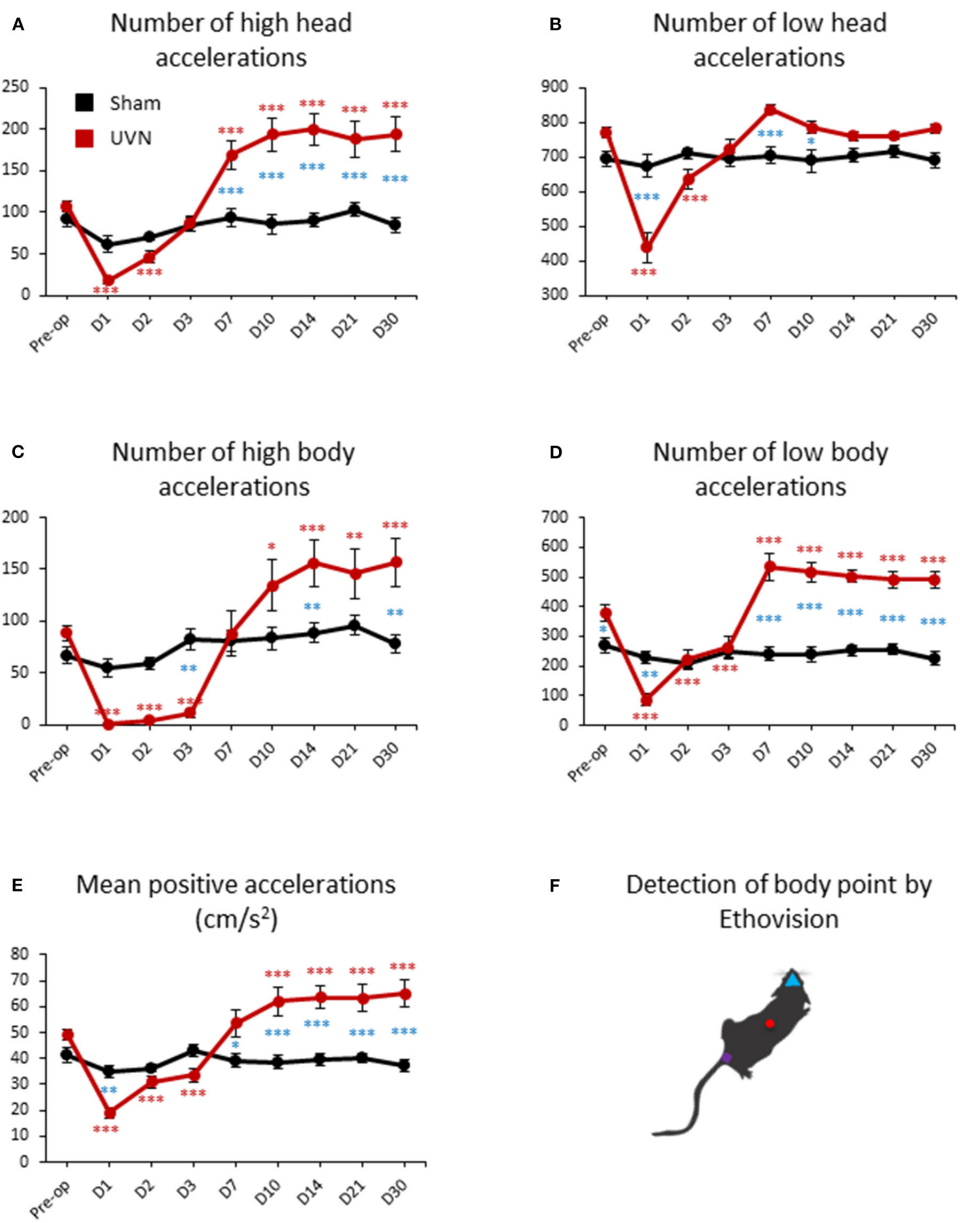
Acceleration of UVN and Sham rats in the open field. **(A)** Curves illustrating the kinetics of the mean number of high acceleration of the nose-point (above 50 cm/s^2^) for UVN (red) and Sham (black) group. **(B)** Curves illustrating the kinetics of the mean number of low acceleration of the nose-point (below 50 cm/s^2^) for UVN (red) and Sham (black) group. **(C)** Curves illustrating the kinetics of the mean number of high acceleration of the center point (above 50 cm/s^2^) for UVN (red) and Sham (black) group. **(D)** Curves illustrating the kinetics of the mean number of low acceleration of the center point (below 50 cm/s^2^) for UVN (red) and Sham (black) group. **(E)** Curves illustrating the mean positive accelerations of the rat (cm/s^2^) for UVN (red) and Sham (black) group. **(F)** Illustration of the detection of body point by Ethovision™ throughout the recordings: nose point (blue triangle), center point (red circle) and tail base (purple square) of each animal. Data represent mean ± SEM; *P < 0.05, **P < 0.01, ***P < 0.001. A significant difference from the pre-operative value is indicated by * in black for the SHAM group. A significant difference from the pre-operative value is indicated by * in red for the UVN group. A significant difference between the SHAM and the UVN group is indicated with * in blue.

Total number of accelerations of the rats can be separated in high (above 50 cm/s^2^) and low (below 50 cm/s^2^) accelerations for both the body and head. UVN rats performed an average of 107.37 ± 5.93 high accelerations (Figure 7A) and 771 ± 15.42 low accelerations (Figure 7B) of the head (calculated from the nose point) before the lesion. High accelerations of the head of UVN rats almost disappeared at day 1 (17.75 ± 4.34, *p* < 0.001), slowly recovered at day 2 (46.12 ± 7.65, *p* < 0.001) and increased significantly from day 7 to day 30 post-lesion (Day 7: 169.25 ± 16.75, *p* < 0.001; Day 10: 193.56 ± 20.11, *p* < 0.001; Day 14: 200 ± 19.17, *p* < 0.001; Day 21: 188.12 ± 21.56, *p* < 0.001; Day 30: 194 ± 20.94, *p* < 0.001; Figure 7A). Low acceleration of the head of UVN rats significantly decreased at day 1 and day 2 post-lesion (Day 1: 438.12 ± 42.12, *p* < 0.001; Day 2: 636.87 ± 27.85, *p* < 0.001) and recovered pre-operative values from day 3 until day 30 (Figure 7B). While the number of high accelerations of the head of the Sham group was significantly lower compared to the UVN group from day 7 to day 30 (Day 7: *p* < 0.001; Day 10: *p* < 0.001; Day 14: *p* < 0.001; Day 21: *p* < 0.001; Day 30: *p* < 0.001; Figure 7A), the number of low accelerations of the head is only significantly different from the UVN group at day 1, day 7 and day 10 (Day 1: *p* < 0.001; Day 7: *p* < 0.001; Day 10: *p* < 0.05; Figure 7B).

UVN rats performed an average of 88.87 ± 7.31 high (Figure 7C) and 379.5 ± 26.56 low body accelerations (Figure 7D) calculated from the center point, before the lesion. High body accelerations almost disappeared during the first 3 days post-lesion (Day 1: 0.87 ± 0.74, *p* < 0.001; Day 2: 4.12 ± 1.49, *p* < 0.001; Day 3: 11.5 ± 4.61, *p* < 0.001) and increased significantly from day 10 to day 30 (Day 10: 134.43 ± 24.33, *p* < 0.001; Day 14: 156.25 ± 22.50, *p* < 0.001; Day 21: 146.12 ± 24.07, *p* < 0.001; Day 30: 156.5 ± 23.66, *p* < 0.001; Figure 7C). Low body accelerations of UVN rats significantly decreased the first 3 days post-lesion (Day 1: 86.12 ± 20.63, *p* < 0.001; Day 2: 221.87 ± 33.06, *p* < 0.001; Day 3: 262.87 ± 38.69, *p* < 0.001) and increased significantly from day 7 until day 30 (Day 7: 533.87 ± 45.80; Day 10: 515.81 ± 34.40, *p* < 0.001; Day 14: 502.5 ± 22.20, *p* < 0.001; Day 21: 490.87 ± 28.40, *p* < 0.001; Day 30: 491.12 ± 29.38, *p* < 0.001; Figure 7D). While the number of high body accelerations of the Sham group significantly differed from those of the UVN group at day 3, 14, and 30 post-lesion (Day 3: *p* < 0.01; Day 14: *p* < 0.01 Day 30: *p* < 0.01; Figure 7C), the number of low body accelerations significantly differed before the lesion and at day 1, 7, 10, 14, 21, and 30 post-lesion (Preop: *p* < 0.05; Day 1: *p* < 0.01 Day 7: *p* < 0.001; Day 10: *p* < 0.001; Day 14: *p* < 0.001; Day 21: *p* < 0.001; Day 30: *p* < 0.001; Figure 7D).

By averaging positive accelerations of UVN animals we found that rats before lesion had a mean acceleration of 49.37 ± 2.04 cm/s^2^ in. We also found the same recovery kinetics after UVN than the frequencies of high body acceleration with two phases: a significant decrease in the mean positive accelerations during the first 3 days after injury (Day 1: 19.08 ± 2.02, *p* < 0.001; Day 2: 30.87 ± 2.32, *p* < 0.001; Day 3: 33.49 ± 2.47, *p* < 0.001) followed by a second phase with an increase from day 10 to day 30 (Day 10: 61.97 ± 5.56, *p* < 0.001; Day 14: 63.42 ± 4.42, *p* < 0.001; Day 21: 63.29 ± 5.07, *p* < 0.001; Day 30: 65.02 ± 5.43, *p* < 0.001; Figure 7E).

All acceleration parameters investigated was not significantly affected by the sham surgery nor postoperative time for the Sham group.

### Posture of UVN and Sham Rats

**Figure 8:**
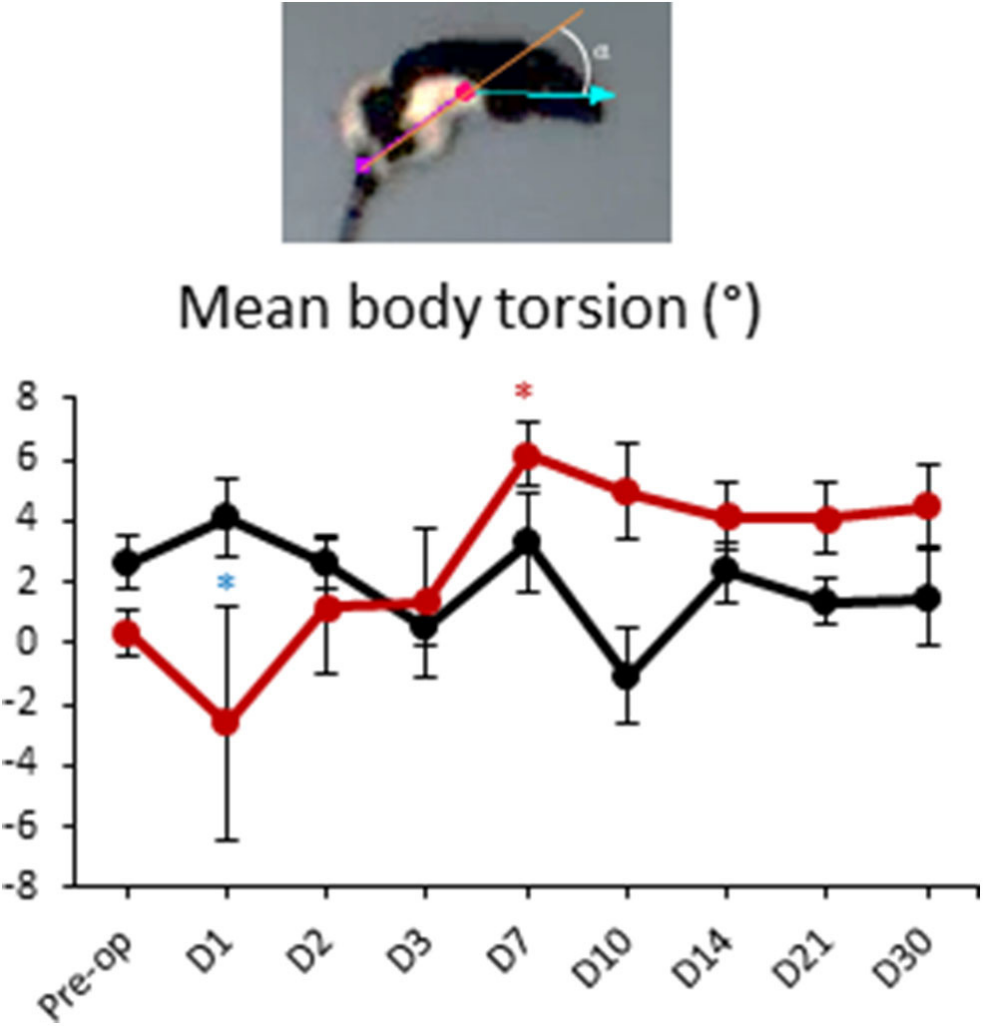
Posture of UVN and Sham rats in the open-field. Curves illustrating the mean body torsion of UVN (red) and Sham (black). Data represent mean ± SEM; *P < 0.05. A significant difference from the pre-operative value is indicated by * in black for the SHAM group. A significant difference from the pre-operative value is indicated by * in red for the UVN group. A significant difference between the SHAM and the UVN group is indicated with * in blue.

Body torsion of the rats before UVN was 0.35° ± 0.74, so the rat's body is straight. UVN produced a significative change in posture on day 7 post-lesion with a mean body torsion of 6.17° ± 1.03 (toward the intact side) compared to pre-operative value (Figure 8), from day 7 to day 30 the mean body torsion was maintained between 4 and 4.9 without significant effect. One day after the surgery UVN rats bent their body significantly on the ipsilesional side compared to the Sham group (Day 1: *p* < 0.05).

The mean body torsion was not significantly affected by the sham surgery nor by the postoperative time for the Sham group.

## Discussion

### Locomotion, Exploration, and Velocity of UVN Rats

Our data showed a significant decrease in the total distance moved and the body velocity during the first 3 days after unilateral vestibular neurectomy (UVN). This phase was followed by a significant increase from day 7 or 10 until day 30 in the distance moved and body velocity, respectively. We demonstrated a significant increase in the meander (sinuous trajectory of the animal) only the first day post-UVN. Certain parameters, such as velocity, were compensated within 7 days post-UVN. These results corroborate those of Lindner et al. (29) in a chemical model of unilateral labyrinthectomy. In this model, rats demonstrated a significant increase in their velocity above baseline after day 7.

Locomotion is an automated behavior generated at the spinal level by the central pattern generator of locomotion (15). However, in response to external factors, the nervous system updates the spinal pattern generator based on multisensory feedback, such as visual, somatosensory or vestibular inputs. In addition to purely vestibular information, vestibular nucleus neurons also receive visual and somatosensory inputs (15, 31), which are required for postural and locomotion control (12, 32). The vestibular nuclei influence motoneurons in the spinal cord through two descending tracts: the medial vestibulospinal and, in prime position to influence the locomotor pattern, the lateral vestibulospinal tract (33). Indeed, the activity of Deiter's neurons is modulated during treadmill locomotion in the cats (34).

Our results from an animal model of vestibular pathology align with observations obtained in vestibular patients subjected to the same type of vestibular deafferentation. Indeed, curative unilateral vestibular neurotomy in vestibular defect patients affects both gait and walking performance and reduces the mean walking velocity (35, 36). These functional parameters are also affected in patients with vestibular disorders (37–40). The slow walking speed of vestibular defect patients can be explained by the reduction in the sensation of imbalance induced by the gain asymmetry of the vestibulo-ocular reflex (VOR) and thus may be a behavioral strategy employed by the subjects to avoid imbalance and fall.

Regarding the velocity parameter, we also demonstrated a time recovery difference in UVN animals between mean and maximum body velocity: the maximum body velocity of the animals had recovered at day 10, compared a recovery to baseline for the mean body velocity at day 7, followed by an increase above the baseline level after day 7. We can assume that after vestibular loss, the priority of the animal is first to move correctly at a medium speed before being able to move faster. In fact, the higher the speed of head movement, the stronger the asymmetry felt in the vestibulo-ocular and vestibulo-spinal pathways. How can the increase in body velocity between day 10 and day 30 post-UVN be explained? Inspired by the observation of a dog with acute unilateral vestibulopathy, Brandt et al. (37) demonstrated that patients with acute vestibulopathy run with less deviation to the affected side than during walking. They suggested that when running, the automatic spinal locomotor program suppresses destabilizing vestibular input. This compensatory behavior may be an option in vestibular defect rats. One possibility is that the rats increase their walking velocity to stabilize their balance (observed between day 10 and day 30). This strategy of increasing walk speed is found in cycling practice, where at low speed, the balance is more precarious than at high speed. If vestibular patients do not run, it is because they consider that their condition probably does not allow them to; it is an avoidance strategy. Indeed, the vestibular contribution to gait variability, which is a predictive marker for falls, declines with faster walking and running (41). Therefore, their balance would be better when running because dynamic balance strategies are different at low and high speeds (41–45).

Regarding the distance moved parameter, we demonstrated an increase exploration of the UVN rats between day 7 and day 30 after the lesion in line with the increase in body velocity. We have two interpretations to these data:

1. The mathematical explanation with a simple formula “Speed = Distance / Time”, informs us that if animals increase their speed without changing the analysis time (10 min) or mobility time (Figure 4), then an increase in velocity is correlated with an increase in distance traveled.
2. An alternative explanation for the increased exploration in the open field may also be due to a spatial disorientation. The head maps (Figure 3) show a higher probability of being in the center of the open field for UVN rats (especially on day 7 to 14 post UVN), which is a kind of non-physiological behavior for rats. Spatial disorientation has been described after unilateral vestibular lesions (46–48) and could explain the increased exploration observed in UVN rats.

### Head Velocity

Interestingly, we found in rats that the mean head velocity recovered faster than the mean walking velocity (day 3 for the head and day 7 for the body velocity). When we looked at the maximum velocity, this recovery was even more obvious: the maximum head velocity was not altered the first days after the vestibular lesion, while maximum walking velocity was impaired and only recovered 10 days after UVN. According to our data, rats, unlike humans, are able to maintain high head velocities during the first few days after UVN while the vestibular syndrome is at its peak. In humans, unlike in rats, the eye has a fovea. A shift of the image onto the fovea is a source of visual discomfort known as oscillopsia. Vestibular patients tend to hold their head still in order to maintain the stability of the visual scene and therefore strongly decrease the speed of head movements in order to avoid this discomfort (49). This behavior is supported by the inability of humans to maintain equilibrium with unstable vision. For head velocities >100°/s, ocular fixation is no longer sufficient to stabilize the image and the VOR takes over. However, unilateral vestibular impairment systematically leads to an alteration of the VOR, causing oscillopsia and thus, instability of the visual scene. Living animals without fovea, such as rats (50), also have the ability to fix and stabilize the eye and have far fewer saccades of the eye than humans. It can be assumed that they are also much less disturbed by image shifts on the retina and thus at oscillopsia. Thus, in the rat, it is not the visual syndrome that is predominant but the posturo-locomotor syndrome. In the rat, retinal slip is not very problematic, and rats can mobilize the head sooner than the body. This behavior obviously requires the implementation of more complex substitution strategies because of the necessity to stabilize its center of gravity as well. We assume that the priority for the animal is first to stabilize its head (and therefore its velocity) before stabilizing its body locomotion. Indeed, when walking in the open field, turns are anticipated by directing the gaze and tilting the head relative to the headings. Eventually, the trunk follows the direction of the head movement (51).

We noticed that the mean head velocity increased between the 3rd and 7th day after UVN, a delay directly linked with the emergence of cephalic nystagmus or “bobbing” (Video 1). Bobbing in rodents with vestibular deficits refers to rapid and abnormal intermittent head and neck sweeping (movements). This is the first time that we automatically quantified this specific bobbing behavior along with the head velocity. During the video analysis of the rat in the open field, we noticed that the head velocity during bobbing was always >100 cm/s. Bobbing in humans is not observed or is poorly documented, but head oscillations are increased after UVN in patients (35). We suggest that bobbing is the result of vestibulo-nucal control asymmetry resulting from vestibulo-colic reflex impairment induced by UVN. Indeed, the axons of the medial vestibulospinal tract that target the motoneurons of the cervical spinal cord contribute to head stabilization (13, 16).

### Acceleration

The head and body accelerations of a rat during free locomotion were quantified for the first time in an automated manner. This measurement is important because it is done under “ecological conditions,” i.e., in spontaneous and not imposed conditions. It is important to remember that the otolith organs, the utricle and saccule, detect linear acceleration and not velocity. We showed that acceleration in UVN rats mimics the kinetics observed for velocity. The kinetics of the acceleration parameters differ depending on whether we are dealing with the head or body segment. The number of high body accelerations (above 50 cm/s^2^) recovers more slowly the first 3 days post-UVN than that of low accelerations (below 50 cm/s^2^). For the velocity, the number of high and low head accelerations recovers differently than the body accelerations. These data are correlated with the displacement profile of UVN rats: if the rats move less during the acute phase of the syndrome (day 1 to day 3 post-UVN), this reduces the number of high and low body accelerations. Furthermore, increases in mean head velocity from day 7 to day 30 related to bobbing behavior are also correlated with an increased number of high head accelerations but not low accelerations at the same postlesional delay. Interestingly, we showed almost a complete disappearance of high accelerations of the body in the first post-lesion days with just a slight increase at day 3 (only 11 high accelerations on day 3 on average compared to 88 in the control condition). This result suggests that it is too difficult for a moving animal to accelerate above 50 cm/s^2^ after the induction of vestibular lesion.

By averaging only the positive accelerations (and thus suppressing decelerations or negative accelerations), we can follow the kinetics of the mean positive acceleration of UVN rats during vestibular compensation. We found that the rats had a mean acceleration of 50 cm/s^2^ when they moved freely before vestibular lesions. For the mean body velocity, the mean acceleration of the UVN rats was reduced until day 7 and then increased above the baseline level after day 7. We also noticed a more important variability from day 7 to day 30 for the mean acceleration and the frequencies of high body accelerations. Since the variation in these parameters was weak before the injury, we can assume that the increase in variability once the animal compensated for the vestibular loss was due to a deficit in acceleration detection. The contralesional utricle and saccule may not be sufficient to properly detect accelerations, resulting in fluctuating accelerations during animal movements. One of the first studies on the perception of linear acceleration in humans reported that thresholds for the detection of linear acceleration in bilateral vestibular loss subjects were nearly 10 times higher than those in control subjects (52). Conversely, Gianna et al. (53) found that the detection of whole-body acceleration in patients with bilateral vestibular loss overlapped with the results from control subjects, but they found high inter-subject variability. On the other hand, the perception of linear acceleration in unilateral labyrinthectomized patients varies according to the orientation of the head (54). The sensitivity of the patient was reduced only when the patient lay with the damaged side downwards.

### Circling and Rotations

First, it is important to differentiate between a specific circling behavior observed after a unilateral vestibular deficit and rotations performed by the animal. The circling behavior induced by a unilateral vestibular deficit is defined when the animal runs in one direction in a circular pattern that occasionally becomes violent (Video 2). Usually, animals with a unilateral vestibular lesion run with a circling behavior in the ipsilesional direction. The term “run” is important here because the circling behavior is faster than a typical rotation performed by the animal (i.e., body axis rotation, Video 3), for example, when he is in a corner and wants to turn back. There is a limit to detecting the rotation of an animal around its own axis when using an automatic system. Some of the rotations detected with Ethovision™ were just rotations made for exploring the environment, while other rotations were linked to the circling behavior caused by the UVN. Therefore, we detected an increase in body axis rotations on the contralesional side (clockwise direction) at day 2 and day 3 for 3 rats, while circling behaviors on the contralesional side of a unilateral vestibular lesion were never observed. Another limitation in the observations of circling behaviors is the influence of the size of the environment in which the animal is analyzed. We noticed that the rats did not exhibit circling behaviors during the first 3 days after UVN in an open field of 80 ×80 cm but did demonstrate circling behaviors in their home cages or in a device of 25 ×25 cm (55). The size of the environment required to observe circling behaviors is therefore important to note for future experiments and discussion of the results.

We demonstrated that rats increased the number of body axis rotations on the ipsilesional side from day 7 to day 30 in correlation with an increase in the number of arena rotations in the same direction. While some body axis rotations detected with video tracking came from rats exhibiting circling behaviors subsequent to UVN, the arena rotations can be interpreted as a bias in the exploratory behavior of the UVN rats. Rats prefer to explore their environment by rotating ipsilaterally to their lesion rather than in the opposite direction. This preferred sense of ipsilesional exploration can be explained by the UVN inducing an asymmetry of muscle tone (56), an increase in weight applied to the ipsilesional paw (55), an ipsilesional shift of their environment on the retina (57), or an ipsilesional path deviation (36). Another possibility comes from a model of vestibular compensation in lampreys (58). The opposite and symmetrical activity of both the left and right groups of reticulospinal neurons allow the lamprey to swim straight. Reticulospinal neurons in the lamprey receive vestibular input from the contralateral side and project to the spinal cord. Thus, unilateral loss of vestibular input causes inactivation of reticulospinal neurons on the contralateral side. This inactivation impairs the symmetrical activity of the left and right groups of reticulospinal neurons and provokes ipsilesional rotation of the lamprey (58).

There is evidence that Parkinson's disease is associated with dysfunction of the vestibular system [for review see Smith (59)]. Eugène et al. (60) studied circling behaviors in vestibular deficient KCNE1 mutant mice and reported that they were associated with increased tyrosine hydroxylase expression—a marker for DA synthesis—in the striatum ipsilateral to the direction of circling. In a model of Parkinson's disease, the lack of dopamine on the injured side leads to rotations by the animal to the ipsilateral side to the lesion. Evidence from animal studies strongly suggests that vestibular input is transmitted to the basal ganglia and to the striatum in particular (59). Thus, circling behaviors could be the result of a striatal electrophysiological imbalance resulting from the electrophysiological imbalance observed in the vestibular nuclei after unilateral vestibular loss. At the behavioral level, unilateral 6-hydroxydopamine lesions cause postural asymmetry (head torsion) and unilateral akinesia in rats. These behavioral disorders are perceptible when intrastriatal dopamine levels are reduced by at least 70% (61, 62). Head torsion and hypotonia are also observed in the UVN model.

The circling behaviors observed in the rodent model after unilateral vestibular loss are not reported in vestibular patients, but there is a deviation of the gait on the lesion side in humans with no full-circle walking. Indeed, the “star walk” or “blind walk” test requires the patient to close his or her eyes and take the same number of steps forward and backward in a straight line several times in a row. Since the deviation in the walk is always in the same direction (and is not corrected by sight since the patient keeps his or her eyes closed), the patient draws a star as he walks (63). When the injury is unilateral, the deviation of the gait is on the lesion side. A similar result can be obtained with the Fukuda test (64).

### Body Tilt and Posture in UVN and Sham Rats

When we analyzed the mean body angle of the animals during the 10 min of video tracking, we noticed that the rats tilted their body on average by 4° on the contralesional side from day 7 and never compensated. X-ray radiography of the guinea pig was performed to investigate the vestibular control on posture of animals with selective lesions on the horizontal and anterior ampullary nerves, the utricular and saccular maculae, and the posterior semicircular canal ampulla (65). Four to seven hours after a unilateral lesion of the horizontal semicircular canal nerve, the authors also found head rotation and bending of the body toward the lesioned side. Interestingly, 3 of the 8 rats in our UVN group bent their body toward the lesioned side by an average of 14° 1 day after the lesion was induced. In our experiment, the mean body tilts were calculated while the rats moved around in the open field and explored their environment, while in Vidal's study, X-ray photographs were taken while the rats were still. Surprisingly, the authors did not notice postural changes with a unilateral anterior canal nerve lesion and proposed that anterior canal afferent information is distributed bilaterally to the spinal cord to describe their results. Unilateral otolith lesions sparing the semicircular canals induced a side tilt of the head-neck ensemble toward the side of the lesion but did not produce inclination about the vertical axis of the body. It appears that cutting the entire vestibular nerve is necessary to induce long-term postural changes in animals.

Trunk orientation in humans after UVN varies if the subject's eyes are open or closed: the trunk deviates toward the operated side until day 90 in the eyes closed condition, and the posture reverses toward the intact side until day 30 in the eyes open condition (35). This postural reversal can be related to modifications in reference frames that the patients base themselves on (66). Since the vertical visual reference of the patient is perceived as tilted toward the intact side, the trunk deviation in the same direction may result from an alignment of the patient's body with respect to the vertical visual reference they perceive.

To explain the inclination of the body on the contralesional side in our UVN rats, we proposed, as in humans, that the inclination of the rat's body on the contralesional side can be related to a subjective vertical deviation of the rat to the operated side. Thus, an inclination of the body on the contralesional side would compensate for the visual vertical reference they perceive. The increase in weight applied on the ipsilateral paws following the lesion from day 7 to day 30 (55) may also be correlated with the inclination of the body on the contralateral side from day 7 to day 30 observed in this study.

### Acute Phase and Compensated Phase of Posturo-Locomotor Symptoms in UVN Rats

The meander, % of time that the animal was immobile or not moving, number of contralesional body axis and arena rotations, mean head and body velocities, maximum body velocity and number of low head accelerations were impaired during the first week after unilateral vestibular lesion and then compensated (i.e., returned to baseline level). However, the distance moved, % of time that the animal was mobile or highly mobile, number of body axis and arena rotations toward the ipsilesional side, mean and maximum head velocity, mean body velocity, bobbing frequencies, number of high head, and body accelerations, number of low body accelerations, mean positive accelerations and mean body torsion on the contralesional side never returned to baseline level. This clearly shows an acute phase (first week post-UVN) of the vestibular syndrome and a compensated phase (after the first week post-UVN) in which some parameters are fully compensated, and others are not. Data from the literature regarding vestibular compensation report that “static deficits (those present in the absence of body movement) fully compensate while dynamic deficits (those present when body is moving) remain poorly compensated” (1, 67). We show in the present study that this assumption is not a dogma since certain parameters, such as maximum body velocity, did not follow this observation. Indeed, the maximum body velocity, which is clearly observable when the body is moving, fully compensated. These original results concerning the kinetics of compensation for static and dynamic vestibular deficits could be due to the use of a fine and automated analysis device.

### Permanent Vestibular Loss Leading to the Expression of an Overcompensated Posturo-Locomotor Phenotype: Neurophysiological Correlates

The restoration of activity in deafferented vestibular nuclei has been shown to contribute to the restoration of vestibular functions (9). Interestingly, the restoration of spontaneous activity in deafferented lateral vestibular nucleus was not complete 4 months after UVN in cats (68) but was completely restored in the same nucleus 1 week after unilateral labyrinthectomy in guinea pigs (69). The authors indicated that this discrepancy could be explained as a difference in species rather than in the nature of the vestibular deafferentation. Given the phylogenetic proximity, the rat could have kinetics of restoration of vestibular nuclei (VN) activity comparable to that of the guinea pig. At the behavioral level, several posturo-locomotor parameters, such as the velocity of the body and the head, return to baseline levels between 7 and 10 days after UVN, exceed their baseline level after this period and reach a plateau that is maintained until 1 month after UVN. This new behavioral profile can be considered a “post-locomotor overcompensated phenotype.”

The return to electrophysiological homeostasis between the two homologous VN is a priority for vestibular functional recovery. It results from both intrinsic mechanisms expressed in deafferented vestibular nuclei and extrinsic mechanisms involving other central nervous system structures, such as a reweighting of other sensory modalities, with an increase in visual, tactile, or proprioceptive weight (70–75). The return to electrophysiological balance in the VN does not reflect the compensation of posturo-locomotor parameters since they significantly exceed their baseline level. These data indicate that in addition to the restoration of spontaneous activity, other mechanisms could explain this “overcompensated behavioral phenotype.” The nature of the evoked activity of the deafferented vestibular nuclei could be implicated. In fact, an increase in the sensitivity of neurons in the VN to dynamic stimuli could explain this overcompensated behavioral phenotype. A change in the sensitivity of neuronal responses to bidirectional rotations has been observed in the vestibular nuclei in cats after unilateral labyrinthectomy (76). This indicates the emergence of a new electrophysiological expression of vestibular nuclei after vestibular loss, probably underlying this “overcompensated behavioral phenotype.” The priority of this new electrophysiological profile within the deafferented vestibular environment is not to restore the vestibular function as it was before the injury but to allow the animal to find a “new” posturo-locomotor balance.

### Clinical Relevance

Balance tests like tandem walking or walking with head turns are not useful for screening people for vestibular impairments but may be useful for assessing vestibular rehabilitation over time among patients with known diagnoses (24). Consistent to Cohen's study and based on our results, it can be assumed that the evaluation of both mean and maximum locomotor velocity could be useful in assessing vestibular compensation in human clinical practice. In view of our results concerning the acceleration parameter, it would also be interesting to take this factor into account in vestibular deficient patients at different stages of the pathology, which could give us information on the level of compensation achieved by the patient. Like we said if vestibular patients do not run, it is because they consider that their condition probably does not allow them to; it is an avoidance strategy. By encouraging them during vestibular rehabilitation to accelerate and increase their locomotor velocity, it is possible that this psychological lock will disappear, and that patients will adapt to a faster walking and a more secure gait. The trajectory of the patient during his displacements can also be evaluated and be similar to what we measure with the “meander.” A possible limitation is the difficulty to have baseline data from the patient prior to vestibular impairment.

## Conclusion

The numerous cellular and molecular rearrangements that take place in the adult vestibular nuclei in the days following permanent unilateral vestibular loss suggest that the vestibular compensation phenomenon recruits plasticity mechanisms similar to those observed during the critical developmental period. Indeed, during a 1-week time window after UVN, there is a an increase in the level of BDNF, a strong cellular proliferation, a glial reaction, and a downregulation of KCC2 associated with an excitatory action of GABA (8, 77, 78). At the behavioral level, impairment and recovery of certain parameters is observed during the same critical 7-day period following UVN. This acute posturo-locomotor phenotype reminds us of the developmental strategies used for walk acquisition. Injury to the adult vestibular system induces a transient reactivation of developmental mechanisms at both the behavioral and cellular levels. This suggests that compensation for gait and postural balance in the vestibulo-lesioned rat is a form of sensorimotor relearning involving both interdependent cellular mechanisms and behavioral strategies.

## Data Availability Statement

The datasets generated for this study are available on request to the corresponding author.

## Ethics Statement

The animal study was reviewed and approved by French Agriculture Ministry Authorization: B13-055-25 Approved by Neurosciences Ethic Committee N°71 from the French National Committee of animal experimentation.

## Author Contributions

GR and BT designed the experiment. GR, EM, NE, AB, ET-D, and DP programmed the experiment. GR, EM, NE, and AB recorded the data. GR and BT analyzed the data. GR, BT, CC, and OD drafted the manuscript. All authors reviewed the manuscript.

## Conflict of Interest

The authors declare that the research was conducted in the absence of any commercial or financial relationships that could be construed as a potential conflict of interest.

## References

[1] Lacour M Helmchen C Vidal PP. Vestibular compensation: the neuro-otologist's best friend. J Neurol. (2016) 263:54–64. 10.1007/s00415-015-7903-4 27083885 PMC4833803

[2] Dutheil S Lacour M Tighilet B. Neurogenic potential of the vestibular nuclei and behavioural recovery time course in the adult cat are governed by the nature of the vestibular damage. PLoS ONE. (2011) 6:e22262. 10.1371/journal.pone.0022262 21853029 PMC3154899

[3] Precht W Shimazu H Markham CH. A mechanism of central compensation of vestibular function following hemilabyrinthectomy. J Neurophysiol. (1966) 29:996–1010. 10.1152/jn.1966.29.6.996 5971666

[4] Darlington CL Smith PF. Molecular mechanisms of recovery from vestibular damage in mammals: recent advances. Prog Neurobiol. (2000) 62:313–25. 10.1016/S0301-0082(00)00002-2 10840152

[5] Darlington CL Dutia MB Smith PF. The contribution of the intrinsic excitability of vestibular nucleus neurons to recovery from vestibular damage: recovery from vestibular damage: role of vestibular nucleus. Eur J Neurosci. (2002) 15:1719–27. 10.1046/j.1460-9568.2002.02024.x 12081651

[6] Dieringer N. Vestibular compensation: neural plasticity and its relations to functional recovery after labyrinthine lesions in frogs and other vertebrates. Prog Neurobiol. (1995) 46:97–129. 10.1016/0301-0082(94)00063-N 7568917

[7] Dutia MB. Mechanisms of vestibular compensation: recent advances. Curr opin Otolaryngol Head Neck. Surg. (2010) 18:420–4. 10.1097/MOO.0b013e32833de71f 20693901

[8] Lacour M Tighilet B. Plastic events in the vestibular nuclei during vestibular compensation: the brain orchestration of a deafferentation code. Restor Neurol Neurosci. (2010) 28:19–35. 10.3233/RNN-2010-0509 20086280

[9] Smith PF Curthoys IS. Mechanisms of recovery following unilateral labyrinthectomy: a review. Brain Res Rev. (1989) 14:155–80. 10.1016/0165-0173(89)90013-1 2665890

[10] Smith PF Darlington CL. Neurochemical mechanisms of recovery from peripheral vestibular lesions (vestibular compensation). Brain Res Rev. (1991) 16:117–33. 10.1016/0165-0173(91)90001-O 1760653

[11] Hain TC Helminski JO Chapter 1: Anatomy and physiology of the normal vestibular system. 3rd ed. In: Herdman SJ, Clendaniel RA, editors. Vestibular Rehabilitation. Philadelphia, PA: F.A. Davis Company (2007). p. 2–18.

[12] Vidal PP Cullen K Curthoys IS Du Lac S Holstein G Idoux E The vestibular system. In: Paxinos G, editor. The Rat Nervous System. London: Elsevier (2015). p. 805–64. 10.1016/B978-0-12-374245-2.00028-0

[13] Wilson VJ Maeda M. Connections between semicircular canals and neck motorneurons in the cat. J Neurophysiol. (1974) 37:346–57. 10.1152/jn.1974.37.2.346 4815209

[14] Wilson VJ Yoshida M. Comparison of effects of stimulation of deiters' nucleus and medial longitudinal fasciculus on neck, forelimb, and hindlimb motoneurons. J Neurophysiol. (1969) 32:743–58. 10.1152/jn.1969.32.5.743 4309026

[15] MacKinnon CD. Sensorimotor anatomy of gait, balance, and falls. In: Handbook of Clinical Neurology. Elsevier (2018). p. 3–26. 10.1016/B978-0-444-63916-5.00001-X PMC7069605 30482322

[16] McCall AA Miller DM Yates BJ. Descending influences on vestibulospinal and vestibulosympathetic reflexes. Front Neurol. (2017) 8:112. 10.3389/fneur.2017.00112 28396651 PMC5366978

[17] Mihailoff GA Haines DE (editors). Chapter 24: Motor system I: peripheral sensory, brainstem, and spinal influence on anterior horn neurons. In: Fundamental Neuroscience for Basic and Clinical Applications. Philadelphia, PA: Elsevier (2018). p. 346–59. 10.1016/B978-0-323-39632-5.00024-4

[18] Navari E Cerchiai N Casani AP. Assessment of vestibulo-ocular reflex gain and catch-up saccades during vestibular rehabilitation. Otol Neurotol. (2018) 39:E1111–7. 10.1097/MAO.0000000000002032 30303945

[19] Deveze A Bernard-Demanze L Xavier F Lavieille J-P Elziere M. Vestibular compensation and vestibular rehabilitation. Current concepts and new trends. Neurophysiol Clin. (2014) 44:49–57. 10.1016/j.neucli.2013.10.138 24502905

[20] Devèze A Montava M Lopez C Lacour M Magnan J Borel L. Vestibular compensation following vestibular neurotomy. Eur Ann Otorhinolaryngol Head Neck Dis. (2015) 132:197–203. 10.1016/j.anorl.2015.04.003 26026684

[21] Visser JE Carpenter MG van der Kooij H Bloem BR. The clinical utility of posturography. Clin Neurophysiol. (2008) 119:2424–36. 10.1016/j.clinph.2008.07.220 18789756

[22] Cohen HS. A review on screening tests for vestibular disorders. J Neurophysiol. (2019) 122:81–92. 10.1152/jn.00819.2018 30995137 PMC6689777

[23] Allum JHJ Scheltinga A Honegger F. The effect of peripheral vestibular recovery on improvements in vestibulo-ocular reflexes and balance control after acute unilateral peripheral vestibular loss. Otol Neurotol. (2017) 38:E531–8. 10.1097/MAO.0000000000001477 29135873

[24] Cohen HS Mulavara AP Peters BT Sangi-Haghpeykar H Bloomberg JJ. Tests of walking balance for screening vestibular disorders. J Vestib Res. (2012) 22:95–104. 10.3233/VES-2012-0443 23000609 PMC3540827

[25] Cohen HS Stitz J Sangi-Haghpeykar H Williams SP Mulavara AP Peters BT. Tandem walking as a quick screening test for vestibular disorders: tandem walking for vestibular screening. Laryngoscope. (2018) 128:1687–91. 10.1002/lary.27022 29226324 PMC5995610

[26] Tramontano M Bergamini E Iosa M Belluscio V Vannozzi G Morone G. Vestibular rehabilitation training in patients with subacute stroke: a preliminary randomized controlled trial. NeuroRehabilitation. (2018) 43:247–54. 10.3233/NRE-182427 30040765

[27] Chen ZP Zhang XY Peng SY Yang ZQ Wang YB Zhang YX. Histamine H1 receptor contributes to vestibular compensation. J Neurosci. (2019) 39:1350–18. 10.1523/JNEUROSCI.1350-18.2018 30413645 PMC6335742

[28] Ito J Nakajima K Mori S. Postnatal changes in locomotor movements after labyrinthectomy in rats. Neurosci Res. (1998) 32:343–7. 10.1016/S0168-0102(98)00101-1 9950061

[29] Lindner M Gosewisch A Eilles E Branner C Krämer A Oos R. Ginkgo biloba extract EGb 761 improves vestibular compensation and modulates cerebral vestibular networks in the rat. Front Neurol. (2019) 10:147. 10.3389/fneur.2019.00147 30858822 PMC6397839

[30] Péricat D Farina A Agavnian-Couquiaud E Chabbert C Tighilet B. Complete and irreversible unilateral vestibular loss: a novel rat model of vestibular pathology. J Neurosci Methods. (2017) 283:83–91. 10.1016/j.jneumeth.2017.04.001 28390798

[31] McCall AA Miller DM DeMayo WM Bourdages GH Yates BJ. Vestibular nucleus neurons respond to hindlimb movement in the conscious cat. J Neurophysiol. (2016) 116:1785–94. 10.1152/jn.00414.2016 27440244 PMC5144685

[32] Niklasson M Tham R Larsby B Eriksson B. The influence of visual and somatosensory input on the vestibulo-oculomotor reflex of pigmented rats. J Vestib Res. (1990) 1:251–62. 1670158

[33] Witts EC Murray AJ Vestibulospinal contributions to mammalian locomotion. Curr Opin Physiol. (2019) 8:56–62. 10.1016/j.cophys.2018.12.010

[34] Orlovsky GN. Activity of vestibulospinal neurons during locomotion. Brain Res. (1972) 46:85–98. 10.1016/0006-8993(72)90007-8 4635375

[35] Borel L Harlay F Magnan J Chays A Lacour M. Deficits and recovery of head and trunk orientation and stabilization after unilateral vestibular loss. Brain. (2002) 125:880–94. 10.1093/brain/awf085 11912120

[36] Borel L Harlay F Lopez C Magnan J Chays A Lacour M. Walking performance of vestibular-defective patients before and after unilateral vestibular neurotomy. Behav Brain Res. (2004) 150:191–200. 10.1016/S0166-4328(03)00257-2 15033292

[37] Brandt T Strupp M Benson J. You are better off running than walking with acute vestibulopathy. Lancet. (1999) 354:746. 10.1016/S0140-6736(99)03179-7 10475195

[38] Buchanan JJ Horak FB. Vestibular loss disrupts control of head and trunk on a sinusoidally moving platform. J Vestib Res. (2001) 11:371–89. 12446963

[39] Cohen HS Sangi-Haghpeykar H. Walking speed and vestibular disorders in a path integration task. Gait Posture. (2011) 33:211–3. 10.1016/j.gaitpost.2010.11.007 21131202 PMC3042484

[40] Mamoto Y Yamamoto K Imai T Tamura M Kubo T. Three-dimensional analysis of human locomotion in normal subjects and patients with vestibular deficiency. Acta Otolaryngol. (2002) 122:495–500. 10.1080/00016480260092282 12206257

[41] Dietrich H Heidger F Schniepp R MacNeilage PR Glasauer S Wuehr M. Head motion predictability explains activity-dependent suppression of vestibular balance control. Sci Rep. (2020) 10:668. 10.1038/s41598-019-57400-z 31959778 PMC6971007

[42] Dietrich H Wuehr M. Strategies for gaze stabilization critically depend on locomotor speed. Neuroscience. (2019) 408:418–29. 10.1016/j.neuroscience.2019.01.025 30703510

[43] Fabre-Adinolfi D Parietti-Winkler C Pierret J Lassalle-Kinic B Frère J. You are better off running than walking revisited: does an acute vestibular imbalance affect muscle synergies?. Hum Mov Sci. (2018) 62:150–60. 10.1016/j.humov.2018.10.010 30384183

[44] Jahn K Strupp M Schneider E Dieterich M Brandt T. Differential effects of vestibular stimulation on walking and running: Neuroreport. (2000) 11:1745–8. 10.1097/00001756-200006050-00029 10852236

[45] MacNeilage PR Glasauer S. Quantification of head movement predictability and implications for suppression of vestibular input during locomotion. Front Comput Neurosci. (2017) 11:47. 10.3389/fncom.2017.00047 28638335 PMC5461342

[46] Jacob P-Y Poucet B Liberge M Save E Sargolini F. Vestibular control of entorhinal cortex activity in spatial navigation. Front Integr Neurosci. (2014) 8:38. 10.3389/fnint.2014.00038 24926239 PMC4046575

[47] Péruch P Borel L Gaunet F Thinus-Blanc G Magnan J Lacour M Spatial performance of unilateral vestibular defective patients in nonvisual versus visual navigation. J Vestib Res. (1999) 9:37–47. 10334015

[48] Zheng Y Darlington CL Smith PF. Impairment and recovery on a food foraging task following unilateral vestibular deafferentation in rats. Hippocampus. (2006) 16:368–78. 10.1002/hipo.20149 16358316

[49] Renga V. Clinical evaluation of patients with vestibular dysfunction. Neurol Res Int. (2019) 2019:3931548. 10.1155/2019/3931548 30863640 PMC6377969

[50] Meier PM Reinagel P. Rats and humans differ in processing collinear visual features. Front Neural Circuits. (2013) 7:197. 10.3389/fncir.2013.00197 24379758 PMC3862114

[51] Imai T Moore ST Raphan T Cohen B. Interaction of the body, head, and eyes during walking and turning. Exp Brain Res. (2001) 136:1–18. 10.1007/s002210000533 11204402

[52] Walsh EG. Role of the vestibular apparatus in the perception of motion on a parallel swing. J Physiol. (1961) 155:506–13. 10.1113/jphysiol.1961.sp006643 13782902 PMC1359871

[53] Gianna C Heimbrand S Gresty M. Thresholds for detection of motion direction during passive lateral whole-body acceleration in normal subjects and patients with bilateral loss of labyrinthine function. Brain Res Bull. (1996) 40:443–7. 10.1016/0361-9230(96)00140-2 8886372

[54] Walsh EG. Perception of linear motion following unilateral labyrinthectomy: variation of threshold according to the orientation of the head. J Physiol. (1960) 153:350–7. 10.1113/jphysiol.1960.sp006538 13782901 PMC1359752

[55] Tighilet B Péricat D Frelat A Cazals Y Rastoldo G Boyer F. Adjustment of the dynamic weight distribution as a sensitive parameter for diagnosis of postural alteration in a rodent model of vestibular deficit. PLoS ONE. (2017) 12:e0187472. 10.1371/journal.pone.0187472 29112981 PMC5675415

[56] Zennou-Azogui Y Borel L Lacour M Ez-Zaher L Ouaknine M Recovery of head postural control following unilateral vestibular neurectomy in the cat: neck muscle activity and neuronal correlates in deiters' nuclei. Acta Otolaryngol. (1993) 113:5–19. 10.3109/00016489309130556 8285044

[57] Halmagyi GM Curthoys IS Cremer PD Henderson CJ Todd MJ Staples MJ. The human horizontal vestibulo-ocular reflex in response to high-acceleration stimulation before and after unilateral vestibular neurectomy. Exp Brain Res. (1990) 81:479–90. 10.1007/BF02423496 2226683

[58] Pavlova EL. Vestibular compensation in lampreys: restoration of symmetry in reticulospinal commands. J Exp Biol. (2004) 207:4595–603. 10.1242/jeb.6247 15579555

[59] Smith PF. Vestibular functions and parkinson's disease. Front Neurol. (2018) 9:1085. 10.3389/fneur.2018.01085 30619045 PMC6297246

[60] Eugène D Deforges S Vibert N Vidal P-P. Vestibular critical period, maturation of central vestibular neurons, and locomotor control. Ann NY Acad Sci. (2009) 1164:180–7. 10.1111/j.1749-6632.2008.03727.x 19645897

[61] Hefti F Melamed E Sahakian BJ Wurtman RJ. Circling behavior in rats with partial, unilateral nigro-striatal lesions: effect of amphetamine, apomorphine, and DOPA. Pharmacol Biochem Behav. (1980) 12:185–8. 10.1016/0091-3057(80)90353-6 7189592

[62] Zigmond MJ Strlcker EM Current topics T. Parkinson's disease studies with an animal model. Life Sci. (1984) 35:5–18. 10.1016/0024-3205(84)90147-4 6146085

[63] Nyabenda A Briart C Deggouj N Gersdorff M A normative study of the vestibulospinal and rotational tests. Adv Physiother. (2004) 6:122–9. 10.1080/14038190310012052

[64] Cohen HS Sangi-Haghpeykar H Ricci NA Kampangkaew J Williamson RA. Utility of stepping, walking, and head impulses for screening patients for vestibular impairments. Otolaryngol Head Neck Surg. (2014) 151:131–6. 10.1177/0194599814527724 24664545 PMC4175306

[65] Vidal PP Wang DH Graf W de Waele C. Chapter 22: Vestibular control of skeletal geometry in the guinea pig: a problem of good trim? In: Allum JHJ, Allum-Mecklenburg DJ, Harris FP, Probst R, editors. Progress in Brain Research. Amsterdam; London; New York, NY; Tokyo: Elsevier (1993). p. 229–43. 10.1016/S0079-6123(08)62282-7 8234750

[66] Borel L Lopez C Péruch P Lacour M. Vestibular syndrome: a change in internal spatial representation. Neurophysiol Clin. (2008) 38:375–89. 10.1016/j.neucli.2008.09.002 19026958

[67] Tighilet B Chabbert C. Adult neurogenesis promotes balance recovery after vestibular loss. Prog Neurobiol. (2019) 174:28–35. 10.1016/j.pneurobio.2019.01.001 30658127

[68] Xerri C Gianni S Manzoni D Pompeiano O. Central compensation of vestibular deficits. I response characteristics of lateral vestibular neurons to roll tilt after ipsilateral labyrinth deafferentation. J Neurophysiol. (1983) 50:428–48. 10.1152/jn.1983.50.2.428 6604136

[69] Ris L de Waele C Serafin M Vidal PP Godaux E. Neuronal activity in the ipsilateral vestibular nucleus following unilateral labyrinthectomy in the alert guinea pig. J Neurophysiol. (1995) 74:2087–99. 10.1152/jn.1995.74.5.2087 8592199

[70] Deliagina T Vestibular compensation in lampreys: impairment and recovery of equilibrium control during locomotion. J Exp Biol. (1997) 200:1459–71. 9319361 10.1242/jeb.200.10.1459

[71] Igarashi M Alford BR Watanabe T Maxian PM. Role of neck proprioceptors for the maintenance of dynamic bodily equilbrium in the squirrel monkey. Laryngoscope. (1969) 79:1713–27. 10.1288/00005537-196910000-00003 4981231

[72] Lacour M Barthelemy J Borel L Magnan J Xerri C Chays A. Sensory strategies in human postural control before and after unilateral vestibular neurotomy. Exp Brain Res. (1997) 115:300–10. 10.1007/PL00005698 9224857

[73] Medendorp WP Alberts BBGT Verhagen WIM Koppen M Selen LPJ. Psychophysical evaluation of sensory reweighting in bilateral vestibulopathy. Front Neurol. (2018) 9:377. 10.3389/fneur.2018.00377 29910766 PMC5992424

[74] Thompson LA Haburcakova C Goodworth AD Lewis RF. An engineering model to test for sensory reweighting: nonhuman primates serve as a model for human postural control and vestibular dysfunction. J Biomech Eng. (2018) 140:011008. 10.1115/1.4038157 29049632 PMC5676644

[75] Tjernström F Fransson P-A Kahlon B Karlberg M Lindberg S Siesjö P Different visual weighting due to fast or slow vestibular deafferentation: before and after schwannoma surgery. Neural Plast. (2019) 2019:1–11. 10.1155/2019/4826238 PMC6398006 30911290

[76] Chan YS Shum DKY Lai CH. Neuronal response sensitivity to bidirectional off-vertical axis rotations: a dimension of imbalance in the bilateral vestibular nuclei of cats after unilateral labyrinthectomy. Neuroscience. (1999) 94:831–43. 10.1016/S0306-4522(99)00374-7 10579574

[77] Dutheil S Watabe I Sadlaoud K Tonetto A Tighilet B. BDNF signaling promotes vestibular compensation by increasing neurogenesis and remodeling the expression of potassium-chloride cotransporter KCC2 and GABAA receptor in the vestibular nuclei. J Neurosci. (2016) 36:6199–212. 10.1523/JNEUROSCI.0945-16.2016 27277799 PMC6604891

[78] Tighilet B Brezun JM Dit Duflo Sylvie G Gaubert C Lacour M. New neurons in the vestibular nuclei complex after unilateral vestibular neurectomy in the adult cat: reactive neurogenesis in adult vestibular lesioned cats. Eur J Neurosci. (2007) 25:47–58. 10.1111/j.1460-9568.2006.05267.x 17241266

