# Corrigendum: Quantitative Evaluation of a New Posturo-Locomotor Phenotype in a Rodent Model of Acute Unilateral Vestibulopathy

**DOI:** 10.3389/fneur.2020.614242

**Published:** 2020-10-26

**Authors:** Guillaume Rastoldo, Emna Marouane, Nada El Mahmoudi, David Péricat, Audrey Bourdet, Elise Timon-David, Olivier Dumas, Christian Chabbert, Brahim Tighilet

**Affiliations:** ^1^Aix Marseille Université-CNRS, Laboratoire de Neurosciences Sensorielles et Cognitives, LNSC UMR 7260, Equipe Physiopathologie et Thérapie des Désordres Vestibulaires, Groupe de Recherche Vertige (GDR#2074), Marseille, France; ^2^Société Française de Kinésithérapie Vestibulaire, Lyon, France

**Keywords:** posture, locomotor activity, vestibular compensation, unilateral vestibular lesion, vestibular syndrome, behavior, ethovision

In the original article, there was a mathematical formula error: “Speed = Distance x Time”

A correction has been made to *section “Discussion”, Sub-section “Locomotion, Exploration, and Velocity of UVN Rats”, Paragraph Number 6*:

- The mathematical explanation with a simple formula “Speed = Distance / Time”, informs us that if animals increase their speed without changing the analysis time (10 min) or mobility time (Figure 4), then an increase in velocity is correlated with an increase in distance traveled.

The authors apologize for this error and state that this does not change the scientific conclusions of the article in any way. The original article has been updated.

